# CDDO-Me Alters the Tumor Microenvironment in Estrogen Receptor Negative Breast Cancer

**DOI:** 10.1038/s41598-020-63482-x

**Published:** 2020-04-16

**Authors:** Michael S. Ball, Rajan Bhandari, Gretel M. Torres, Viktor Martyanov, Mohamed A. ElTanbouly, Kim Archambault, Michael L. Whitfield, Karen T. Liby, Patricia A. Pioli

**Affiliations:** 1 Department of Microbiology and Immunology, Geisel School of Medicine, Lebanon, New Hampshire United States of America; 2 Department of Biomedical Data Science, Geisel School of Medicine, Lebanon, New Hampshire United States of America; 3 Department of Pharmacology and Toxicology, Michigan State University, East Lansing, Michigan United States of America

**Keywords:** Cancer, Immunology

## Abstract

The tumor microenvironment (TME) is an essential contributor to the development and progression of malignancy. Within the TME, tumor associated macrophages (TAMs) mediate angiogenesis, metastasis, and immunosuppression, which inhibits infiltration of tumor-specific cytotoxic CD8+ T cells. In previous work, we demonstrated that the synthetic triterpenoid CDDO-methyl ester (CDDO-Me) converts breast TAMs from a tumor-promoting to a tumor-inhibiting activation state *in vitro*. We show now that CDDO-Me remodels the breast TME, redirecting TAM activation and T cell tumor infiltration *in vivo*. We demonstrate that CDDO-Me significantly attenuates IL-10 and VEGF expression but stimulates TNF production, and reduces surface expression of CD206 and CD115, markers of immunosuppressive TAMs. CDDO-Me treatment redirects the TAM transcriptional profile, inducing signaling pathways associated with immune stimulation, and inhibits TAM tumor infiltration, consistent with decreased expression of CCL2. In CDDO-Me-treated mice, both the absolute number and proportion of splenic CD4^+^ T cells were reduced, while the proportion of CD8^+^ T cells was significantly increased in both tumors and spleen. Moreover, mice fed CDDO-Me demonstrated significant reductions in numbers of CD4^+^ Foxp3^+^ regulatory T cells within tumors. These results demonstrate for the first time that CDDO-Me relieves immunosuppression in the breast TME and unleashes host adaptive anti-tumor immunity.

## Introduction

The tumor microenvironment (TME) provides the interface for communication between malignant and immune cells. As cells accumulate genetic and epigenetic aberrations, they recruit immune and other cellular mediators that collectively establish chronic inflammation in the TME. Mounting evidence demonstrates that interaction between tumor cells and immune cells in the TME facilitates tumor immune evasion by redirecting immune cell activation from a state of immune stimulation to immune suppression.

The activation state and effector function of tumor-associated lymphoid and myeloid cells are profoundly influenced by the local TME, and these cells are likely an essential component of most tumors, regardless of tumorigenic trigger^1,2^. Tumor associated macrophages (TAMs) are key orchestrators of tumor cell survival and metastasis and shape adaptive immune responses via interaction with CD4^+^ and CD8^+^ T cell populations, among others^3^. TAMs recruit monocytes to the TME by secreting chemokines including CCL2, and promote angiogenesis through production of vascular endothelial growth factor (VEGF)^4,5^, transforming growth factor β (TGF-β) and matrix metalloproteinases (MMPs)^6^. Immunosuppression in the TME is mediated by TAMs through production of TGF-β, IL-10, and arginase 1^7^, which inhibit T cell activation and survival.

TAMs can account for up to 50% of the tumor mass in breast cancer and genetic depletion of TAMs results in delayed tumor progression^8^, indicating that these cells play a non-redundant role. High TAM volumes are associated with poor clinical outcome for several solid tumor types, including cancer of the breast, bladder, prostate, cervix, and ovary^9,10^. Because these cells support tumor growth, progression and metastatic potential, TAMs are an attractive therapeutic target. As macrophage activation can be altered by micro-environmental cues, recent studies have used multiple approaches, including recombinant cytokine and anti-cytokine therapies, to modulate TAM activity *in vivo*. For example, CSF therapies have been used clinically because of their inherent immunomodulatory functions, but these may be associated with severe adverse side-effects^11^. Thus, there is a clear need for the development of safe and effective therapeutics to modulate TAM activation.

In this regard, we recently demonstrated that the methyl ester of the synthetic triterpenoid 2-cyano-3,12-dixooleana-1,9(11)dien-28-oic acid (CDDO-Me) repolarizes alternatively activated human macrophages and murine primary mammary TAMs from immunosuppressive to immunostimulatory^12^. CDDO-Me has been shown to strongly induce Nrf2 activation via Keap1 interaction and to upregulate anti-inflammatory and antioxidant pathways in cancer cells and cell lines^13,14^. In this regard, nanomolar concentrations of CDDO-Me have been shown to inhibit inducible nitric oxide synthase (iNOS) and reactive oxygen species (ROS) production in cells that have been stimulated with inflammatory cytokines. In addition to Nrf2, CDDO-Me interacts with other signaling proteins, including IkappaB alpha kinase beta (IKKβ), signal transducer and activator of transcription 3 (STAT3), human epidermal growth factor receptor 2 (HER2), and mammalian target of rapamycin (mTOR)^15^. CDDO-Me is pleiotropic, as low doses confer protection against inflammation, whereas high doses induce cytotoxic, antiproliferative effects^13^. Thus, CDDO-Me may inhibit carcinogenesis through multiple mechanisms: by attenuation of inflammation in the TME and by direct killing of cancer cells by apoptosis. Indeed, studies using mouse models of lung, prostate, and breast cancer have demonstrated that CDDO-Me acts as both a chemo-preventative and chemotherapeutic agent^13^. Although the effects of CDDO-Me on tumor cell activation have been widely characterized, there are few reports describing the ability of this drug to modulate leukocyte activation in the TME^13^.

We now report that CDDO-Me inhibits TAM tumor infiltration and redirects TAM activation *in vivo*. Consistent with previous results, CDDO-Me treatment inhibits TAM infiltration to mammary tumors, and decreases TAM expression of the macrophage chemo-attractant CCL2. CDDO-Me significantly inhibits expression of IL-10 and VEGF and increases production of TNF. Microarray results demonstrate that CDDO-Me treated TAMs adopt an immunostimulatory transcriptional profile, upregulating several signaling pathways including TNFα/NFκB, type 1 and type 2 interferon response, and STAT5 signaling. Notably, TAM production of CXCL16, which recruits activated T cells^16^, is significantly enhanced by CDDO-Me. CDDO-Me increases the ratio of CD8^+^:CD4^+^ T cells in tumors, and in the spleen, CD8^+^ T cell numbers are enhanced concurrent with decreases in CD4^+^ T cells. These results demonstrate for the first time the *in vivo* immunological effect of CDDO-Me on the breast TME and implicate TAMs as potential effectors of drug response through repolarization.

## Results

### CDDO-Me alters breast tumor TAM infiltration and activation *in vivo*

**Figure 1:**
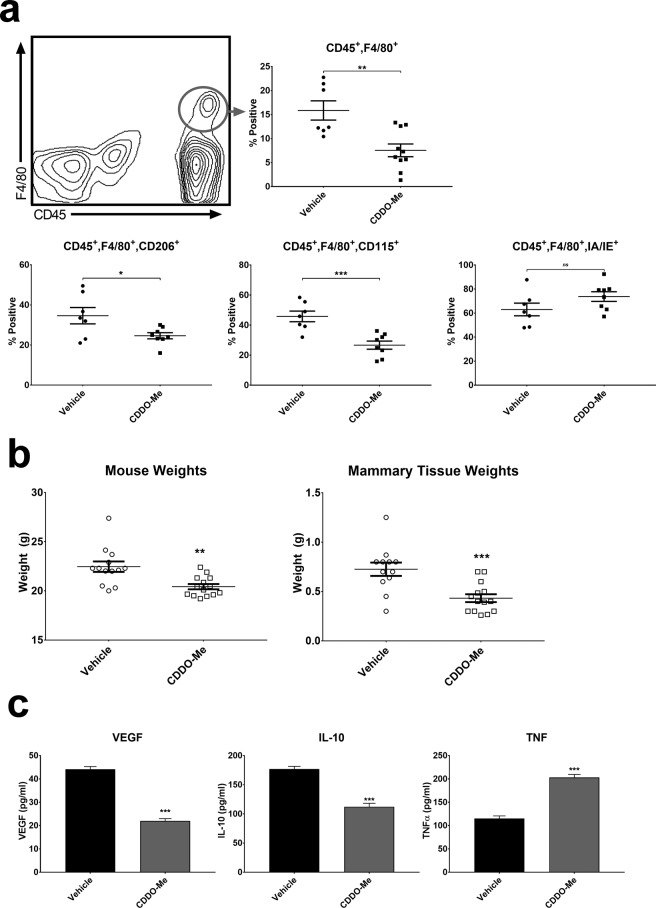
*CDDO-Me redirects cytokine production of F4/80*^+^ *TAMs in vivo*. (**a**) Whole mammary/tumor tissue of PyMT mice fed diet with or without 50 mg CDDO-Me/kg diet for 8 weeks was digested to a single cell suspension and analyzed by multicolor flow cytometry for analysis of macrophages. Expression of surface markers is represented as percentage of cells positive for the respective marker vs. vehicle (*n* = *6*–*10 per group*). (**b**) Mouse weights were recorded immediately prior to tissue resection and mammary/tumor tissue weights were recorded immediately after tissue resection (*n* = *12*–*14 per group)*. (**c**) TAMs were isolated using F4/80^+^ magnetic bead selection and cultured in complete DMEM for 24 hours. Culture supernatants were analyzed by ELISA for murine VEGF, IL-10, and TNF-α (*n* = *3 per group)*. *p < 0.05, **p < 0.01, ***p < 0.005 vs. vehicle; ns = nonsignificant. Cytokine data are representative of 3 biologically independent experiments.

In published work, we have shown that *in vitro* treatment of TAMs and human M(CSF-1) macrophages with CDDO-Me redirects macrophage activation from immunosuppressive to immunostimulatory^12^. To determine the effect of CDDO-Me on TAM activation *in vivo*, PyMT^+/−^ female mice were fed control diet or diet containing CDDO-Me (50 mg/kg diet) beginning at 4 weeks of age. Because maximal TAM infiltration to mammary glands and tumors of PyMT mice is observed at 12 weeks^17^, mice were euthanized after 8 weeks on diet, and mammary glands and tumors were analyzed by multicolor flow cytometry. Both mammary glands and tumors were analyzed together, as many PyMT mice do not possess palpable tumors at or before 12 weeks of age. However, microscopic lesions are present in mammary glands at 12 weeks^17^. Consistent with a previous report^17^, 15% fewer F4/80^+^ TAMs were detected in the mammary tissue and tumors of mice fed CDDO-Me diet compared with mice fed control diet (Fig. 1a). Notably, we observed that the mice that received CDDO-Me weighed significantly less than their control counterparts, and that the weight of their total mammary and tumor tissue was also significantly reduced (Fig. 1b). We observed no significant difference in the rate of diet consumption between mice fed CDDO-Me diet compared with mice fed control diet, and mice that received CDDO-Me gained weight at a consistent rate and were observed to be as active and healthy as their control counterparts. This suggests that reduced mass is not the result of increased fasting or morbidity.

As demonstrated in Fig. 1a, CDDO-Me significantly reduced the numbers of TAMs that express two surface markers characteristic of immunosuppressive macrophages, CD206 and CD115. The macrophage mannose receptor CD206 is a C-type lectin receptor that is a marker of pro-angiogenic macrophages^18^, and expression of CD115, the CSF-1 receptor (CSF1R) is associated with advanced breast cancer progression and cancer mortality^19^. Moreover, CD115 has been shown to be important for the accumulation of F4/80^+^ TAMs and overall cancer progression in the PyMT model^20,21^. In accordance with their role in immune suppression, TAMs have poor antigen presenting capabilities and downregulate MHC Class II expression^22,23^. However, CDDO-Me treatment significantly increased TAM surface expression of IA/IE (Fig. 1a), consistent with the induction of immune activation. Following overnight culture, TAM supernatants were harvested and analyzed by ELISA for expression of soluble VEGF, IL-10, and TNF-α proteins as shown in fig. 1c. TAMs isolated from mice on diet with CDDO-Me showed significantly attenuated levels (up to 50%) of pro-angiogenic VEGF and immunosuppressive IL-10. Consistent with *in vitro* results^12^, CDDO-Me also increased TAM expression of the pro-inflammatory protein TNF-α by two-fold.

### CDDO-Me confers immunostimulatory transcriptional activation profile to breast TAMs

**Figure 2:**
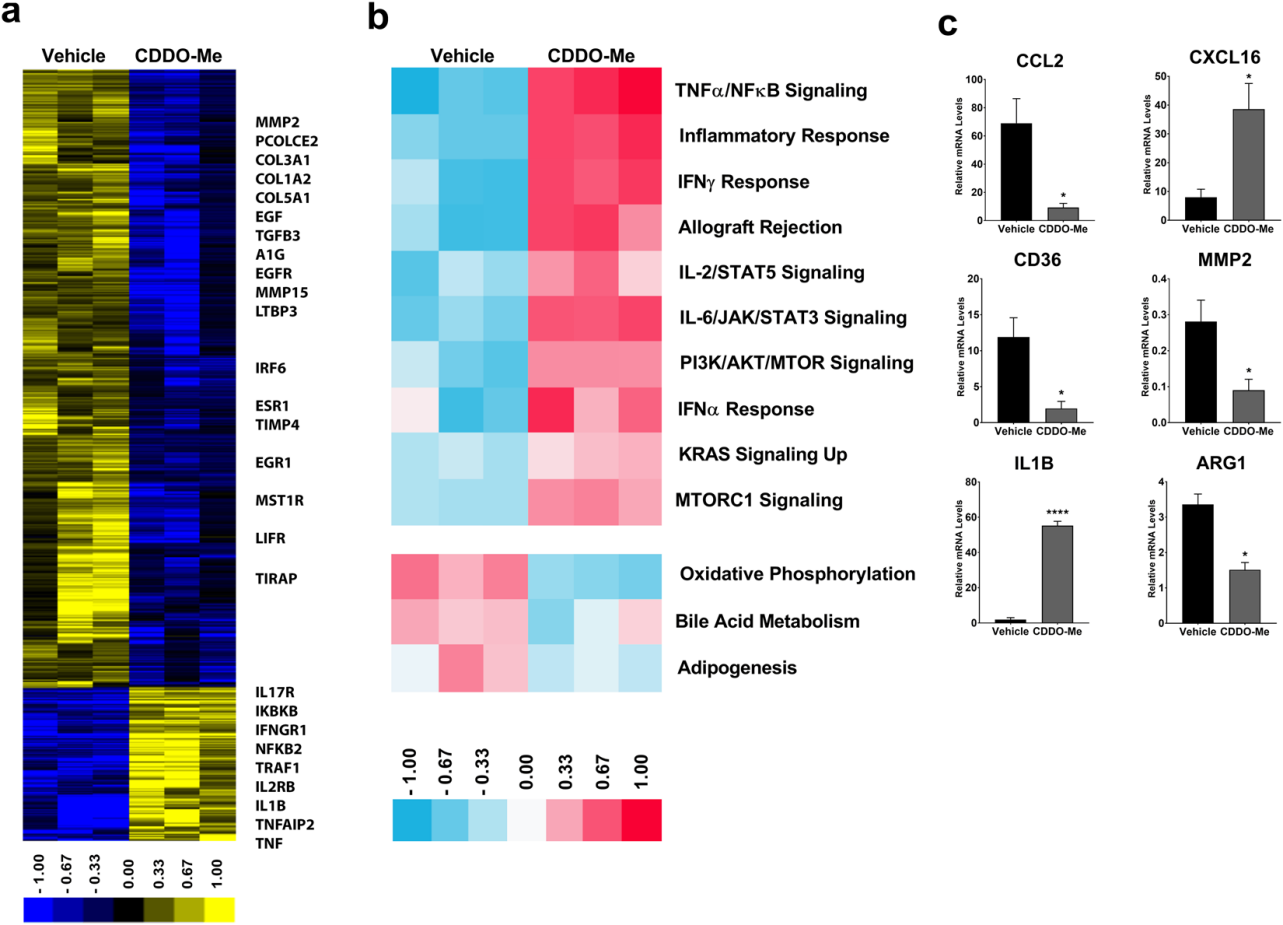
*CDDO-Me alters transcriptional program of TAMs in vivo*. F4/80^+^ TAMs were isolated from the mammary tissue of 12 week old mice that were fed control or CDDO-Me diet (50 mg/kg diet) for 8 weeks. (**a**) Total RNA was isolated and analyzed by microarray. (**b**) Hallmark pathway analysis of differentially expressed genes (DEGs) in microarray was performed. (**c**) Select mRNA transcripts were validated by Taqman qRT-PCR. *p < 0.05, **p < 0.01, ***p < 0.005 vs. vehicle. Microarray data and qRT-PCR represent the average of three independent experiments.

To determine the gene expression profile elicited by *in vivo* CDDO-Me-treatment, we performed microarray analysis on TAMs (Fig. 2a) isolated from CDDO-Me-treated mice. We identified 1285 significant differentially expressed genes (DEGs) in TAMs using SAM (unpaired *t* test, SAM FDR < 5%; complete list of genes available online in Supplemental Tables S1 and S2). This analysis demonstrated that 255 genes were upregulated in CDDO-Me-treated TAMs compared with untreated controls. Using GSEA, we identified 10 Hallmark pathways that were significantly increased by *in vivo* treatment of TAMs with CDDO-Me (FDR ≤ 5%), which included signaling pathways involved in mediating immune activation, such as TNFα and NFκB signaling, type I and type II interferon responses, and pro-inflammatory responses (Fig. 2b). Increased expression of these pathways is characteristic of macrophages that promote immune activation (reviewed in^24^). We also ran GSEA for our expression data set vs. the Canonical Pathways database (Supplemental Fig. S1), which revealed additional upregulated pathways in CDDO-Me treated TAMs, including those that regulate IL-12 signaling, NFκB signal transduction, TLR cascade, and TNFR1 signaling. In contrast, 1,030 genes were significantly upregulated in TAMs of mice fed control chow compared with CDDO-Me-treated mice, and control TAMs were enriched for transcripts that regulate lipid metabolism and extracellular matrix remodeling. As it is known that alternatively activated macrophages and TAMs consume more oxygen and have a greater reliance on oxidative phosphorylation for energy synthesis^25^ compared with classically activated macrophages, these results further reinforce the ability of CDDO-Me to induce an immune-activated, tumor-inhibitory activation profile in TAMs *in vivo*.

**Figure 3:**
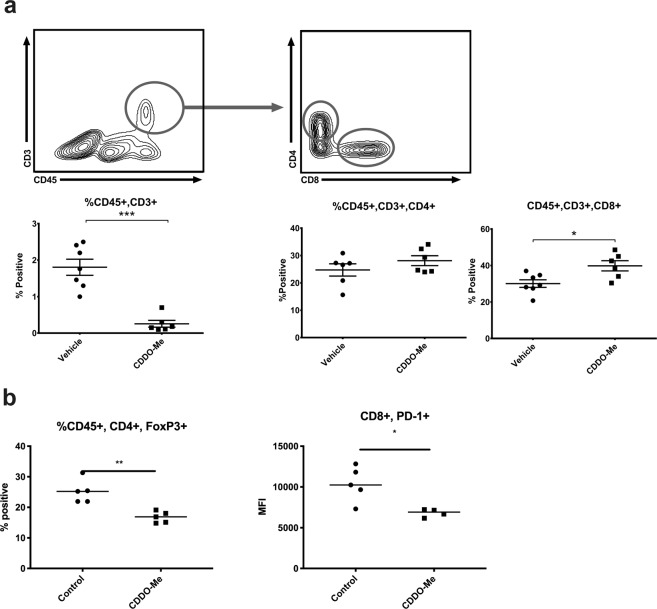
*CDDO-Me increases the proportion of CD8*^+^ *T cells and reduces Treg numbers in tumors in vivo*. Whole mammary/tumor tissue of mice fed diet with or without 50 mg/kg CDDO-Me for 8 weeks was digested to a single cell suspension and analyzed by multicolor flow cytometry for analysis of CD4 + and CD8+ T cell populations (**a**). Dual positive CD4/Foxp3 T cell percentages were compared in control and CDDO-Me-treated mice (**b**). Expression of PD1 on CD8+ T cells was assessed using mean fluorescence intensity (MFI) (**b**). Where indicated, expression of surface markers is represented as percentage of cells positive for the respective marker *(n* = *5–10 per group)*. *p < 0.05, **p < 0.01, ***p < 0.005 vs. vehicle.

**Figure 4:**
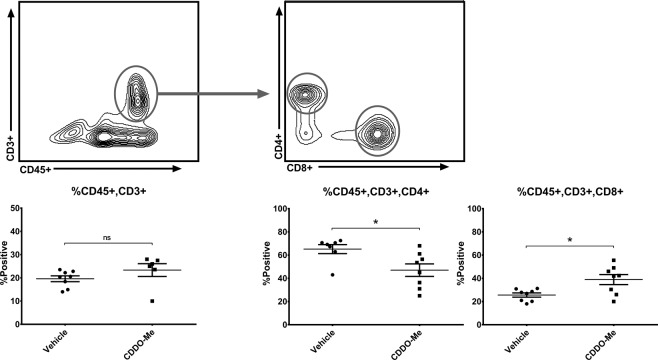
*CDDO-Me decreases splenic CD4*+ *and augments CD*8+ *T cell populations in vivo*. Spleens from mice fed control or 50 mg/kg CDDO-Me diet for 8 weeks was digested to a single cell suspension and analyzed by multicolor flow cytometry for analysis of T cell populations. Expression of surface markers is represented as percentage of cells positive for the respective marker *(n* = *6–8 per group)*. *p < 0.05 vs. vehicle.

We next performed qRT-PCR analysis on several key transcripts known to be important indicators of macrophage function and polarization that were identified by the array and from prior *in vitro* studies (Fig. 2c). CCL2 is a potent chemoattractant for blood monocytes, and is necessary for the recruitment of myeloid cells to the TME^26^. As seen in Fig. 2c, expression of CCL2 is significantly decreased, suggesting reduced expression of this chemokine mediates, at least in part, the attenuation of TAM tumor infiltration observed in Fig. 1a. In contrast, expression of the CD8^+^ T cell chemoattractant CXCL16 was significantly upregulated in TAMs by *in vivo* CDDO-Me treatment, paralleling changes observed in T cell recruitment (Figs. 3 and 4). Consistent with redirection of TAM activation from tumor-promoting to tumor-inhibiting, expression of CD36, which is upregulated on immunosuppressive breast TAMs^27^, was significantly attenuated by CDDO-Me (Fig. 2c). Because MMP2 has been implicated in metastatic breast disease^28^, we assessed *in vivo* CDDO-Me effects on expression of this metalloproteinase. As demonstrated in Fig. 2c, CDDO-Me treatment decreased MMP2 levels in TAMs. Moreover, CDDO-Me significantly attenuated expression of Arg1 in TAMs treated *in vivo*, as previously observed *in vitro*^12^. Increased levels of Arg1 are characteristic of alternatively activated macrophages and immunosuppressive TAMs. mRNA levels of IL1β, which mediates T cell activation, were also significantly increased by *in vivo* CDDO-Me treatment.

### T cell populations are modulated in the breast TME by CDDO-Me

Because tumor infiltrating lymphocytes (TILs) are important in the assessment and outcomes of immunotherapeutic intervention and are useful prognostic indicators for many cancers, the T cell repertoire of CDDO-Me-treated tumors was interrogated using multicolor flow cytometry. CD3^+^ T cells were reduced by 50% in mammary tissue and tumors of mice fed CDDO-Me compared with controls (Fig. 3a). Notably in human breast cancer, higher baseline proportion of all CD3^+^ populations is associated with subtypes with a poor prognosis and hormone receptor negativity^29^.

While the proportion of CD3^+^ cells that were CD4^+^ did not change, the percentage of CD3^+^ cells that were positive for the cytotoxic T cell marker CD8 significantly increased, which is a positive prognostic indicator in human disease^10^. Notably, administration of CDDO-Me resulted in attenuation of surface PD1 levels on CD8^+^ T cells (Fig. 3b), suggesting relief of exhaustion^30^.

We next interrogated whether CD4^+^ T cell subtypes were altered by drug administration, as these cells have been implicated as important regulators of both pro and anti-tumor immunity. In this regard, Foxp3-expressing regulatory T cells (Tregs), which constitute a significant proportion of total CD4^+^ tumor-infiltrating T cells, facilitate tumor immune evasion, and ablation of Tregs leads to significant decreases in both primary and metastatic tumor burden^31^. As demonstrated in Fig. 3b, *in vivo* CDDO-Me treatment significantly inhibited Treg tumor infiltration, but drug treatment did not alter Th2 or Th17 tumor T cell subsets (online in Supplemental Fig. S2).

### CDDO-Me alters T cell populations in the spleen

To determine the potential effect of CDDO-Me on immune populations systemically, we next measured T cell populations within the spleen as shown in Fig. 4. Mice fed CDDO-Me did not exhibit a significant change in total splenic CD3^+^ cells, but did show a significant decrease (15%) in CD3^+^CD4^+^ cells, concurrent with a significant increase in CD3^+^CD8^+^ cells. There were no statistically significant differences among CD3^+^CD4^+^ subtypes (online in Supplemental Fig. S3).

## Discussion

Given the significant contribution of the TME to the development and progression of malignancy^32^, increased efforts are focused on generating therapies to target this niche. Because we identified CDDO-Me as an immune-modulator in a previous study^12^, we hypothesized that *in vivo* administration of this drug would alter immune activation within the breast TME. This report demonstrates for the first time the ability of CDDO-Me to redirect TAM activation and T cell tumor infiltration in the TME of PyMT mice. In accordance with *in vitro* findings, we showed that CDDO-Me reprograms breast TAMs *in vivo* from tumor-promoting to tumor-inhibiting as determined by surface phenotype, cytokine production, and transcriptional profile. In addition, our results demonstrated that the TME of CDDO-Me-treated PyMT mice contains lower overall CD3^+^ T cell populations and reduced numbers of CD4^+^ FoxP3^+^ T cells, but higher proportions of CD8^+^ T cells. Relative levels of CD8^+^ T cells were also elevated in the spleen concurrent with a decrease in CD4^+^ T cells. It is likely that CDDO-Me-mediated alterations in TAM activation result in both direct and indirect effects on T cell recruitment and activation.

In this regard, CDDO-Me significantly increased TNF and inhibited VEGF and IL-10 production in TAMs (Fig. 1). Attenuated expression of IL-10 may alleviate immunosuppression of other myeloid effector cells, as TAM-derived IL-10 has been shown to inhibit IL-12 production in intratumoral dendritic cells, leading to suppression of anti-tumor CD8^+^ T cell activation^33^. In this model, decreased IL-10 levels from CDDO-Me-treated TAMs results in enhanced activation of CD8 effectors. Because we also demonstrated that CDDO-Me increased the proportion of CD8^+^ T cells in tumors (Fig. 3a), this may have significant effects on inhibition of tumor growth. Furthermore, CDDO-Me may enhance antigen presentation within the TME through inhibition of IL-10, as IL-10 suppresses dendritic cell but increases macrophage differentiation^34^. CDDO-Me-mediated effects on TAM IL-10 production may be both direct and indirect, as expression of CCL2 was also significantly blunted by drug treatment in both TAMs (Fig. 2) and tumor cells^17^. Prior work has shown that CCL2 induces IL-10 production in alternatively activated macrophages^35^.

In addition to regulation of IL-10 expression, inhibited CCL2 production in TAMs likely contributes to the reduction in TAM infiltration observed in this study (Fig. 2) and others^17^. As demonstrated by Franklin *et al*., TAMs differentiate from inflammatory CCR2^+^ blood monocytes, and CCL2 is required for this recruitment, independent of tissue resident mammary macrophages^26^. CCL2-mediated TAM recruitment is critical for the progression and malignant conversion of mammary tumors in the PyMT model^36^. Therefore, disruption of the CCL2/TAM axis may be an important component of the mechanism by which CDDO-Me inhibits tumor progression in this model.

Our results show that CDDO-Me treatment modulated both myeloid and T cell tumor infiltration. CDDO-Me-mediated reductions in myeloid tumor cell numbers are likely attributable to significantly attenuated expression of CCL2, IL-10, and VEGF, which also regulate monocyte recruitment to TAMs^37^. One potential mediator of altered T cell recruitment is CXCL16, which is chemotactic for activated invariant natural killer T cells (iNKT), NK cells, and effector and activated CD8^+^ T cells. CXCL16 expression is a favorable prognostic indicator in colorectal, renal, and breast cancer^38^, and recruits effector CD8^+^ T cells following *in vivo* treatment with ionizing radiation and anti-CTLA-4 therapy in murine models of breast cancer^16,39^. Microarray and qRT-PCR analysis of TAMs demonstrated significant increases in CXCL16 mRNA expression following *in vivo* treatment with CDDO-Me (Fig. 2). These findings suggest that TAMs treated with CDDO-Me *in vivo* may drive the enhanced recruitment of CD8^+^ T cells to the PyMT mammary TME observed in this study.

Intriguingly, several pathways involved in the regulation of T cell recruitment and activation are upregulated in CDDO-Me treated TAMs, including activation of IFNγ and TNF signaling pathways, IL1 and IL12 mediated signaling, class II expression, lymphocyte interaction, and adaptive immunity (Fig. 2). As demonstrated by ELISA, CDDO-Me-treated TAMs express more TNF and less IL-10, which can directly impact the expansion and activation of T cells *in vivo*^40^. TAMs treated with CDDO-Me also produce less arginase (*Arg1*) and more IL-1β (*Il1b*) transcript. Because arginase inhibits cytotoxic effector T cell responses^41^ and IL-1β promotes antigen-specific T cell maturation and proliferation^42^, CDDO-Me may facilitate CD8 T^+^ cell activation in the TME. In further support of this, we demonstrated that CDDO-Me significantly reduced surface expression of PD1 on CD8^+^ T cells. As recent work has shown the therapeutic benefit of targeted inhibition of PD1/PDL1 signaling in triple negative breast cancer patients^2^, CDDO-Me may provide an additional tool for countering checkpoint inhibition.

*In vivo* administration of CDDO-Me significantly reduced tumor Treg numbers, and it is also possible that CDDO-Me inhibits Treg activity and expansion. TNF and IL-1β, which are induced in TAMs by CDDO-Me treatment (Figs. 1 and 2), may attenuate suppression mediated by Tregs^43^. Because IL-10 enhances tumoral neuropilin-1 (nrp-1) Treg signaling, which regulates nTreg immunosuppressive functions^12,44^, CDDO-Me inhibition of IL-10 production may provide an additional mechanism of not only limiting Treg recruitment to tumors but also of reducing Treg signal transduction, thereby attenuating immunosuppression in the TME.

While TAMs are critical in regulating the growth of primary tumors, they are also important mediators of metastasis. As demonstrated in microarray results, expression of matrix metalloproteinases (MMPs) 2 and 15 was significantly decreased in TAMs by CDDO-Me treatment. These MMPs are known to be involved in the remodeling of the TME extracellular environment^45,46^ and can enhance metastasis in several solid tumors^28,47^. Interestingly, tissue inhibitor of metalloproteinase 2 (TIMP2) was also attenuated by treatment. TIMPs are the natural inhibitors of MMPs, and while TIMP2 can directly inhibit MMP2, it is also responsible for the activation of active MMP2 *in vivo*^48^. Although beyond the scope of the current study, it will important to define the contribution of CDDO-Me to metastatic development and progression.

Because CDDO-Me is multifunctional, effects on immune cell activation are likely mediated by a variety of signaling pathways. In ongoing studies, we have shown that CDDO-Me inhibits activation of STAT3 and NFκB signaling pathways in TAMs. Notably, each of these pathways has been implicated in the regulation of macrophage polarization. Indeed, CDDO-Me has also been shown to target cyclin D1, EGFR, and STAT3 signaling in PyMT tumor cells^17^. As we have shown that CDDO-Me induces immune activation in the TME, it may provide an important adjuvant therapy. In this regard, nanoparticle delivery of CDDO-Me enhances the efficacy of vaccine therapy for the treatment of melanoma^49^. Consistent with our findings, CDDO-Me alleviates immunosuppression and enhances CD8^+^ T cell tumor infiltration in this model^49^. These results coupled with our own report implicate CDDO-Me as an immunotherapeutic for breast, melanoma, and potentially other types of cancer. The effects of CDDO-Me on multiple cell types and signaling pathways likely contribute to its therapeutic efficacy in the PyMT model. Studies in our laboratory are now focused on defining the mechanisms of action of CDDO-Me and in identifying potential combination treatments to maximize therapeutic efficacy.

## Methods

### Mice

Mice were handled ethically according to the Regulations for the Management of Laboratory Animals at the Geisel School of Medicine at Dartmouth. The experimental protocol for the ethical use of these animals was approved by the Institutional Animal Care and Use Committee at the Geisel School of Medicine at Dartmouth (protocol 00002010(m1)). Female heterozygous mice expressing the polyomavirus middle T-antigen (PyMT) under the control of the MMTV promoter on C57BL/6J background were obtained from Dr. Jeffrey Pollard (Albert Einstein College of Medicine, Bronx, NY) and genotyped as previously described^50^. Mice were euthanized by inhalation of carbon dioxide followed by cervical dislocation. All efforts were made to minimize animal suffering.

### *In Vivo* drug treatments

CDDO-Me was synthesized as described^51^. Four-week-old female PyMT mice were fed powdered 5002 rodent chow (PMI Feeds) or 5002 powdered diet containing CDDO-Me (50 mg/kg diet). To determine the effects of CDDO-Me on the mammary TME, PyMT +/− female mice were fed either control diet or CDDO-Me in diet from 4 weeks of age until 12 weeks of age. After 8 weeks on diet, mammary glands were harvested and tissue was processed for F4/80 bead selection or whole tissue flow cytometric analysis. At time of euthanasia, mice and mammary glands were weighed.

### Isolation of primary PyMT tumor associated macrophages (TAMs) and tumor cells

Mammary tissue was removed from 12-week-old female PyMT mice and incubated in digestion media, which consisted of an enzyme mixture of collagenase (300 U/ml, Sigma), dispase (1 U/ml, Worthington), and DNAse (2 U/ml, Calbiochem), for 45 minutes at 37 °C with stirring. Cells were then passed through 70 μm and 40 μm Cell Strainers (BD Falcon), followed by incubation with biotinylated F4/80 antibody (clone: BM8, eBioscience) and a subsequent 15-minute incubation with magnetic streptavidin-coated beads (Miltenyi Biotec). Cells were washed between incubations with PBS (+2 mM EDTA). Total F4/80^+^ positive mouse macrophages were isolated according to the manufacturer’s specifications (Miltenyi Biotec) in PBS (+2 mM EDTA and 0.5% FBS). Positively selected TAMs and negative flow-through fractions were immuno-phenotyped using flow cytometry to evaluate surface marker expression and to ensure purity of TAM isolation. TAM expression of CD11b and F4/80 was confirmed by flow cytometry. Primary PyMT tumor cells from female PyMT^+/−^ mice were isolated as described by Tran *et al*.^17^. Briefly, mammary tissue was resected and digested as above, filtered through a 40 μm strainer, centrifuged at 220 × *g* for 10 minutes, and plated in DMEM +10% FBS for two days prior to RNA isolation.

### Isolation of splenocytes

Spleens were homogenized mechanically, then filtered through 70 μm and 40 μm Cell Strainers. Splenocytes were subsequently washed with PBS (+2 mM EDTA) before staining with T cell subset antibodies for flow cytometry.

### Cell culture & reagents

Primary mouse TAMs, tumor cells, and splenocytes were cultured in DMEM (4 mM L-glutamine, 4500 mg/L glucose) supplemented with 10% FBS, 0.25 M HEPES, and 100 μg/ml penicillin streptomycin. Cells were rested overnight post-isolation and prior to subsequent processing.

### RNA extraction and cDNA synthesis

Total RNA from mouse cells was obtained using the miRNeasy Mini Kit (Qiagen) per manufacturer’s instructions. Complementary DNA (cDNA) was synthesized from 100 ng total RNA and random hexamers using the SuperScript III First-Strand Synthesis System (Life Technologies)^12^.

### Quantitative real time PCR (qRT-PCR)

Quantitative real time PCR was performed using TaqMan Probe single tube assays (Life Technologies) for mouse (*Ccl2*, *Cxcl16, Arg1*, *Il1b, Cd36*, and *Mmp2*) genes. The StepOnePlus Real-Time PCR System (Applied Biosystems) was used for amplification and detection. Threshold cycle number was determined using Opticon software. mRNA levels were normalized to β−actin, which control studies showed is not altered by CDDO-Me treatment, using the equation 2^−(Et-Rt)^, where Rt is the mean cycle threshold for the control gene and Et is the mean threshold for the experimental gene. Thermal cycling conditions for qRT-PCR consisted of an initial incubation at 50 °C for 2 min and 95 °C for 10 min, followed by 40 cycles of 95 °C for 15 sec and 60 °C for 1 min. Product accumulation was measured during the extension phase and all samples were run in triplicate^12^.

### Microarray hybridization

Total RNA was extracted from mammary TAMs or tumor cells from PyMT mice fed CDDO-Me or control chow for 8 weeks using the miRNeasy kit (Qiagen). RNA integrity and quantification were determined using a 4200 TapeStation (Agilent). Samples were amplified and labeled using an Agilent Low Input Quick Amp Labeling Kit and were hybridized against Universal Mouse Reference (Stratagene) to Agilent Whole Mouse Genome DNA microarrays (G4852A) in a common reference-based design as previously described^52^. Agilent Feature Extraction Image Analysis Software (Version 10.7.3) was utilized for the extraction of data in raw microarray image files.

### Microarray data processing and analysis

Mouse expression data were lowess-normalized log_2_ Cy5:Cy3 ratios, and filtered for intensity:background ratio of ≥1.5-fold in 1 or both channels and for which at least 80% of the data passed this threshold. This resulted in 6406 probes selected for analysis, which were collapsed to 5897 gene symbols. Expression values were multiplied by −1 to convert them to log2(Cy3/Cy5) ratios.

Microarray data were pre-processed and analyzed as in Long *et al*.^52^. For control vs. CDDO-Me treatment comparisons, differentially expressed genes (DEGs) were identified using Significance Analysis of Microarrays (SAM^53^) with two class unpaired response variable and False Discovery Rate (FDR) correction of 5%. Pathways with significant changes in expression were identified by gene set enrichment analysis (GSEA)^54,55^ using the GenePattern module. All GSEA analyses were corrected for multiple hypothesis testing. GSEA were run against the Canonical Pathways database comprising gene sets from several pathway databases as well as the Hallmarks molecular signatures database^56^. For GSEA, the ‘permutation type’ parameter was set to ‘gene set’. ssGSEA enrichment scores were normalized by dividing by the maximum ssGSEA enrichment score for this expression dataset. Normalized ssGSEA enrichment scores for significant pathways (false discovery rate (FDR) < 5%) were extracted, clustered and visualized as described above for the expression data.

### Flow cytometry

All fluorophore-conjugated antibodies were obtained from Biolegend: anti-CD45-FITC, anti-CD206-PE, anti-F4/80-PE/Cy7, anti-CD115-APC, anit-IA/IE-APC/Cy7, anti-CD3-BV421, anti-FoxP3-FITC, anti-CD45-PE, anti-CD4-PE/Cy7, anti-RoRγt-PE/Cy7, anti-CD8a-APC/Cy7, anti-Granzyme B-FITC, anti-PD-1-APC, and anti-CD11b-BV421. Cell staining was performed for 1 hour at 4 °C, with 2% rat serum (Sigma) to reduce antibody binding to Fc receptors. In all conditions, doublets and multiplets were excluded by forward scatter pulse width (FSC-W) vs. forward scatter pulse area (FSC-A) gating and live cells were selected via negative selection for Yellow Amine Dye (Life Technologies) staining. Gating of positively stained cells was determined by fluorescence-minus-one (FMO) controls. Cells were analyzed using an 8-color MACSQuant 10 (Miltenyi Biotec) with three laser sources (405 nm, 488 nm, 635 nm) and FlowLogic 501.2 A software (Inivai Technologies).

### Enzyme-linked immunosorbent assay (ELISA)

ELISA kits for quantitation of IL10, VEGF, TNF protein expression in cell-culture supernatants were purchased from R&D systems, and ELISAs were performed according to manufacturer’s protocols.

### Statistical analysis

Figures are representative of three independent experiments as indicated in Figure Legends. All experiments were repeated at least 3 times, unless otherwise noted and at least 3 technical replicates of each analyte were included in each assay. Results are described as mean ± SEM and were analyzed by unpaired student’s t-Test. Significance was achieved at p < 0.05.

## Supplementary information

Supplementary Information.

## Data Availability

Data will be made publicly available and deposited to NCBI GEO.
