# Supplementary material for: CDDO-Me Alters the Tumor Microenvironment in Estrogen Receptor Negative Breast Cancer
Source: Sci Rep. 2020 Apr 16;10:6560. doi: 10.1038/s41598-020-63482-x (PMC7162855; doi:10.1038/s41598-020-63482-x)
Supplement: Supplementary file 1 — Supplementary Information. [file 41598_2020_63482_MOESM1_ESM.pdf]

Supplemental Material (Tables and Figures)

CDDO-Me Alters the Tumor Microenvironment in Estrogen Receptor Negative Breast Cancer

Michael S. Ball[1], Rajan Bhandari[1], Gretel M. Torres[1], Viktor Martyanov[2], Mohamed A. ElTanbouly[1], Kim Archambault[2], Michael L. Whitfield[2], Karen T. Liby[3], and Patricia A. Pioli[1*]

[1]Department of Microbiology and Immunology, Geisel School of Medicine, Lebanon, New Hampshire, United States of America

[2]Department of Biomedical Data Science, Geisel School of Medicine, Hanover, New Hampshire, United States of America

[3]Department of Pharmacology and Toxicology, Michigan State University, East Lansing, Michigan, United States of America

*Corresponding Author: Patricia A. Pioli; One Medical Center Drive; Borwell Building, Room 644E; Lebanon, NH 03756; phone #: 603-650-2584; fax #: 603-650-6130; e-mail: pioli@dartmouth.edu

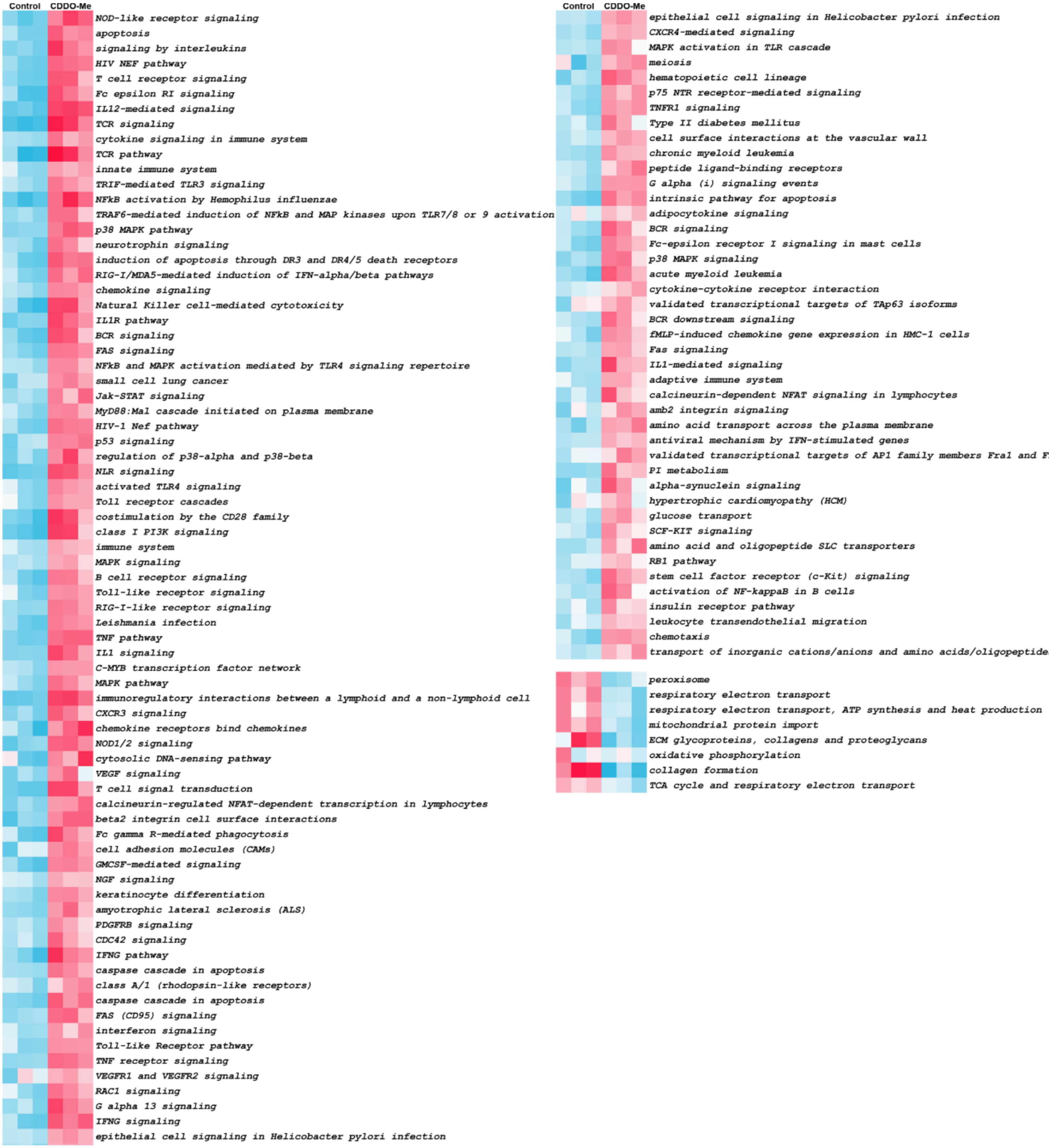

Supplemental Figure S1. *Canonical Pathway Analysis*. F4/80$^+$ TAMs were isolated from the mammary tissue of 12 week old mice that were fed with or without CDDO-Me in diet at 50 mg/kg for 8 weeks. Total RNA was isolated and analyzed by canonical pathway analysis of differentially expressed genes.

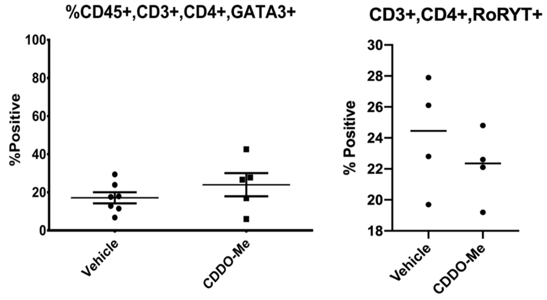

Supplemental Figure S2. *Tumor CD4$^+$ T cell subset analysis*. Whole mammary/tumor tissue of mice fed diet with or without 50 mg/kg CDDO-Me for 8 weeks was digested to a single cell suspension and analyzed by multicolor flow cytometry for analysis of CD4$^+$T cell populations. Dual positive CD4/GATA3 and CD4/RORγ cell percentages were compared in control and CDDO-Me-treated mice. Expression of surface markers is represented as percentage of cells positive for the respective marker *(n = 5-10 per group)*. No significant differences in subsets were observed.

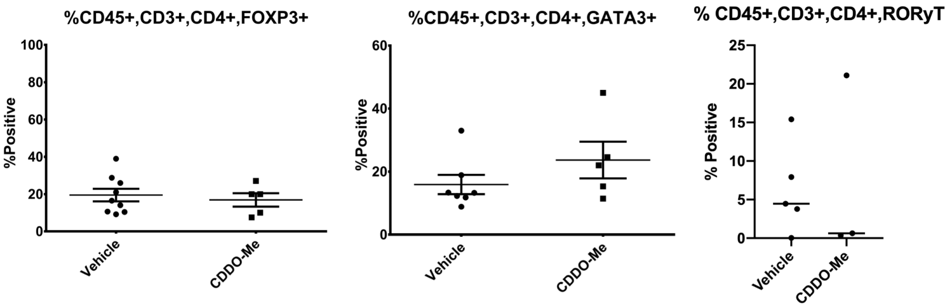

Supplemental Figure S3. *Splenic CD4$^+$ T cell subset analysis*. Whole mammary/tumor tissue of mice fed diet with or without 50 mg/kg CDDO-Me for 8 weeks was digested to a single cell suspension and analyzed by multicolor flow cytometry for analysis of CD4$^+$T cell populations. Dual positive CD4/FOXP3, CD4/GATA3, and CD4/RORγ cell percentages were compared in control and CDDO-Me-treated mice. Expression of surface markers is represented as percentage of cells positive for the respective marker *(n = 5-10 per group)*. No significant differences in subsets were observed.

Supplemental Table S1 **Genes with increased expression (FDR < 5%) in control samples in Figure 2A as determined using Significance Analysis of Microarrays (SAM)**

FDR q-value (%)	Gene symbol	Gene name
0	Tmed3	transmembrane emp24 domain containing 3
0	1700012g19Rik	RIKEN cDNA 1700012G19 gene
0	Mb	myoglobin
0	Clec3b	C-type lectin domain family 3, member b
0	Myh8	myosin, heavy polypeptide 8, skeletal muscle, perinatal
0	Map1lc3a	microtubule-associated protein 1 light chain 3 alpha
0	MGI:891976	muscle, intestine and stomach expression 1
0	P4ha2	procollagen-proline, 2-oxoglutarate 4-dioxygenase (proline 4-hydroxylase), alpha II polypeptide
0	Klk11	kallikrein 11
0	Arhgdig	Rho GDP dissociation inhibitor (GDI) gamma
0	Jagn1	jagunal homolog 1 (Drosophila)
0	D11ertd498e	DNA segment, Chr 11, ERATO Doi 498, expressed
0	Hist1h1c	histone 1, H1c
0.545795608	MGI:2153084	serologically defined colon cancer antigen 33 like
0.545795608	MGI:1351330	cocaine and amphetamine regulated transcript
0.545795608	Fntb	farnesyltransferase, CAAX box, beta
0.545795608	MGI:104966	brain and kidney protein
0.545795608	A030007d23Rik	RIKEN cDNA A030007D23 gene
0.545795608	4930506l13Rik	RIKEN cDNA 4930506L13 gene
0.545795608	Tmem41b	transmembrane protein 41B
0.545795608	Loxl2	lysyl oxidase-like 2
0.545795608	Actn3	actinin alpha 3
0.545795608	Wnt4	wingless-related MMTV integration site 4
0.545795608	Myoz1	myozenin 1
0.545795608	Ick	intestinal cell kinase
0.545795608	Nr2f6	nuclear receptor subfamily 2, group F, member 6
0.545795608	Egf	epidermal growth factor
0.545795608	Ppm1j	protein phosphatase 1J
0.545795608	Snrpn	small nuclear ribonucleoprotein N
0.735307972	4732486j07Rik	RIKEN cDNA 4732486J07 gene
0.735307972	Rab17	RAB17, member RAS oncogene family
0.735307972	Ubox5	U box domain containing 5
0.735307972	Smug1	single-strand selective monofunctional uracil DNA glycosylase
0.735307972	Ppp1r3c	protein phosphatase 1, regulatory (inhibitor) subunit 3C
0.735307972	Capn5	calpain 5
0.735307972	Kcnk1	potassium channel, subfamily K, member 1
0.860848358	Ckm	creatine kinase, muscle
0.860848358	C1qtnf4	C1q and tumor necrosis factor related protein 4
0.860848358	B3gnt6	UDP-GlcNAc:betaGal beta-1,3-N-acetylglucosaminyltransferase 6
0.860848358	Exosc4	exosome component 4
0.860848358	Cited4	Cbp/p300-interacting transactivator, with Glu/Asp-rich carboxy-terminal domain, 4
0.860848358	Syngr1	synaptogyrin 1
0.860848358	B930093c12Rik	RIKEN cDNA B930093C12 gene
0.860848358	2700038n03Rik	RIKEN cDNA 2700038N03 gene
0.860848358	Copz2	coatomer protein complex, subunit zeta 2
0.860848358	Narg1l	NMDA receptor regulated 1-like
0.860848358	Cd248	CD248 antigen, endosialin
1.011633898	2310016a09Rik	RIKEN cDNA 2310016A09 gene
1.011633898	Pthr1	parathyroid hormone receptor 1
1.011633898	Ogn	osteoglycin
1.011633898	Klc3	kinesin light chain 3
1.011633898	MGI:2674071	protein containing single MORN motif in testis
1.011633898	Zswim1	zinc finger, SWIM domain containing 1
1.011633898	Rtn2	reticulon 2 (Z-band associated protein)
1.011633898	Thop1	thimet oligopeptidase 1
1.011633898	Aldh1a7	aldehyde dehydrogenase family 1, subfamily A7
1.011633898	1700025k23Rik	RIKEN cDNA 1700025K23 gene
1.011633898	Mgst3	microsomal glutathione S-transferase 3
1.011633898	Kdelr3	KDEL (Lys-Asp-Glu-Leu) endoplasmic reticulum protein retention receptor 3
1.011633898	Wfs1	Wolfram syndrome 1 homolog (human)
1.011633898	Wisp2	WNT1 inducible signaling pathway protein 2
1.011633898	Cyhr1	cysteine and histidine rich 1
1.011633898	Islr	immunoglobulin superfamily containing leucine-rich repeat
1.011633898	Fos	FBJ osteosarcoma oncogene
1.157205989	9330140g23	hypothetical protein 9330140G23
1.157205989	Gprc5b	G protein-coupled receptor, family C, group 5, member B
1.157205989	Pet112l	PET112-like (yeast)
1.157205989	Spa17	sperm autoantigenic protein 17
1.157205989	Smo	smoothened homolog (Drosophila)
1.157205989	1700065o13Rik	RIKEN cDNA 1700065O13 gene
1.157205989	Zfp637	zinc finger protein 637
1.157205989	5430428g01Rik	RIKEN cDNA 5430428G01 gene
1.157205989	Fbxw8	F-box and WD-40 domain protein 8
1.157205989	3010021m21Rik	RIKEN cDNA 3010021M21 gene
1.157205989	Serpine2	serine (or cysteine) proteinase inhibitor, clade E, member 2
1.157205989	Rab3d	RAB3D, member RAS oncogene family
1.157205989	2810432l12Rik	RIKEN cDNA 2810432L12 gene
1.157205989	0710008k08Rik	RIKEN cDNA 0710008K08 gene
1.157205989	1700027n10Rik	RIKEN cDNA 1700027N10 gene
1.157205989	Pxmp4	peroxisomal membrane protein 4
1.242774037	Psmd5	proteasome (prosome, macropain) 26S subunit, non-ATPase, 5
1.242774037	Irs1	insulin receptor substrate 1
1.242774037	1700088e04Rik	RIKEN cDNA 1700088E04 gene
1.242774037	Cops7a	COP9 (constitutive photomorphogenic) homolog, subunit 7a (Arabidopsis thaliana)
1.242774037	Coq4	coenzyme Q4 homolog (yeast)

1.242774037	Fgf10	fibroblast growth factor 10
1.242774037	2810417j12Rik	RIKEN cDNA 2810417J12 gene
1.242774037	Ddx1	DEAD (Asp-Glu-Ala-Asp) box polypeptide 1
1.242774037	Oact1	O-acyltransferase (membrane bound) domain containing 1
1.242774037	Btc	betacellulin, epidermal growth factor family member
1.242774037	4931406o17Rik	RIKEN cDNA 4931406O17 gene
1.242774037	Zdhhc2	zinc finger, DHHC domain containing 2
1.242774037	Myoc	myocilin
1.242774037	Ai467484	expressed sequence AI467484
1.242774037	Serpinf1	serine (or cysteine) proteinase inhibitor, clade F, member 1
1.242774037	Crtap	cartilage associated protein
1.242774037	Gna11	guanine nucleotide binding protein, alpha 11
1.242774037	Sirt5	sirtuin 5 (silent mating type information regulation 2 homolog) 5 (S. cerevisiae)
1.242774037	Rab2	RAB2, member RAS oncogene family
1.242774037	Zfp41	zinc finger protein 41
1.242774037	Rpn1	ribophorin I
1.242774037	Cd164	CD164 antigen
1.242774037	D9ertd280e	DNA segment, Chr 9, ERATO Doi 280, expressed
1.242774037	Pde9a	phosphodiesterase 9A
1.242774037	Tde2l	tumor differentially expressed 2-like
1.242774037	Mamdc2	MAM domain containing 2
1.242774037	Zfp35	zinc finger protein 35
1.242774037	Asb8	ankyrin repeat and SOCS box-containing protein 8
1.242774037	Tmem9	transmembrane protein 9
1.242774037	Kdelc2	KDEL (Lys-Asp-Glu-Leu) containing 2
1.242774037	Pccb	propionyl Coenzyme A carboxylase, beta polypeptide
1.242774037	Ggt1	gamma-glutamyltransferase 1
1.242774037	Plekhf1	pleckstrin homology domain containing, family F (with FYVE domain) member 1
1.242774037	Mgc102251	similar to regulator of sex-limitation candidate 11
1.242774037	Tmem15	transmembrane protein 15
1.242774037	4933412h03Rik	RIKEN cDNA 4933412H03 gene
1.242774037	Kcnn4	potassium intermediate/small conductance calcium-activated channel, subfamily N, member 4
1.298581626	Hspa12b	heat shock protein 12B
1.298581626	Myl1	myosin, light polypeptide 1
1.298581626	Cox6a2	cytochrome c oxidase, subunit VI a, polypeptide 2
1.298581626	MGI:3028594	zinc finger protein 422, related sequence 1
1.298581626	Stard10	START domain containing 10
1.298581626	Ai646023	expressed sequence AI646023
1.298581626	Cyp2b9	cytochrome P450, family 2, subfamily b, polypeptide 9
1.298581626	Aw209491	expressed sequence AW209491
1.298581626	4632408a20Rik	RIKEN cDNA 4632408A20 gene
1.298581626	0610011f06Rik	RIKEN cDNA 0610011F06 gene
1.298581626	Gm237	gene model 237, (NCBI)
1.298581626	Bc029169	cDNA sequence BC029169
1.298581626	Gabrd	gamma-aminobutyric acid (GABA-A) receptor, subunit delta
1.298581626	2400009b11Rik	RIKEN cDNA 2400009B11 gene
1.298581626	Coq7	demethyl-Q 7
1.298581626	Pkia	protein kinase inhibitor, alpha
1.298581626	1700022c21Rik	RIKEN cDNA 1700022C21 gene
1.298581626	Map3k7ip1	mitogen-activated protein kinase kinase kinase 7 interacting protein 1
1.298581626	Ntn1	netrin 1
1.298581626	6330416l07Rik	RIKEN cDNA 6330416L07 gene
1.298581626	Egln3	EGL nine homolog 3 (C. elegans)
1.298581626	Tmem4	transmembrane protein 4
1.298581626	Slc35f3	solute carrier family 35, member F3
1.298581626	Ntf5	neurotrophin 5
1.298581626	Apoa1bp	apolipoprotein A-I binding protein
1.298581626	Efs	embryonal Fyn-associated substrate
1.298581626	2810430m08Rik	RIKEN cDNA 2810430M08 gene
1.298581626	Cdv1	carnitine deficiency-associated gene expressed in ventricle 1
1.298581626	Bpgm	2,3-bisphosphoglycerate mutase
1.298581626	1110038f14Rik	RIKEN cDNA 1110038F14 gene
1.298581626	5730449l18Rik	RIKEN cDNA 5730449L18 gene
1.298581626	Bc055368	cDNA sequence BC055368
1.557122764	Bc025546	cDNA sequence BC025546
1.557122764	4930430j20Rik	RIKEN cDNA 4930430J20 gene
1.557122764	Lman1	lectin, mannose-binding, 1
1.557122764	Trp63	transformation related protein 63
1.557122764	4930467b06Rik	RIKEN cDNA 4930467B06 gene
1.557122764	Thra	thyroid hormone receptor alpha
1.557122764	Grtp1	GH regulated TBC protein 1
1.557122764	1700019d03Rik	RIKEN cDNA 1700019D03 gene
1.557122764	Mrps31	mitochondrial ribosomal protein S31
1.557122764	1300017k07Rik	RIKEN cDNA 1300017K07 gene
1.557122764	Abcd3	ATP-binding cassette, sub-family D (ALD), member 3
1.557122764	Jrk	jerky
1.557122764	4930506d23Rik	RIKEN cDNA 4930506D23 gene
1.557122764	Tst	thiosulfate sulfurtransferase, mitochondrial
1.557122764	Stc2	stanniocalcin 2
1.557122764	Itga11	integrin, alpha 11
1.557122764	Tbl2	transducin (beta)-like 2
1.557122764	4933424n09Rik	RIKEN cDNA 4933424N09 gene
1.557122764	Decr2	2-4-dienoyl-Coenzyme A reductase 2, peroxisomal
1.557122764	1110025i09Rik	RIKEN cDNA 1110025I09 gene
1.557122764	Esr1	estrogen receptor 1 (alpha)
1.557122764	Gstm5	glutathione S-transferase, mu 5
1.557122764	Ranbp6	RAN binding protein 6

1.557122764	Txndc2	thioredoxin domain containing 2 (spermatozoa)
1.557122764	Tpd52l1	tumor protein D52-like 1
1.557122764	Ndufb9	NADH dehydrogenase (ubiquinone) 1 beta subcomplex, 9
1.557122764	Timm17a	translocator of inner mitochondrial membrane 17a
1.557122764	Thap4	THAP domain containing 4
1.557122764	Trim68	tripartite motif containing 68
1.557122764	2410081m15Rik	RIKEN cDNA 2410081M15 gene
1.557122764	2310020a21Rik	RIKEN cDNA 2310020A21 gene
1.557122764	Usp46	ubiquitin specific protease 46
1.557122764	C1qtnf3	C1q and tumor necrosis factor related protein 3
1.557122764	Tmcc2	transmembrane and coiled-coil domains 2
1.557122764	Bmpr1a	bone morphogenetic protein receptor, type 1A
1.557122764	1700021f05Rik	RIKEN cDNA 1700021F05 gene
1.557122764	Adamts2	a disintegrin-like and metalloprotease (reprolysin type) with thrombospondin type 1 motif, 2
1.557122764	Hyal2	hyaluronidase 2
1.557122764	Apln	apelin
1.557122764	Bc021608	cDNA sequence BC021608
1.557122764	Tgfb3	transforming growth factor, beta 3
1.557122764	Qtrt1	queuine tRNA-ribosyltransferase 1
1.557122764	Grem2	gremlin 2 homolog, cysteine knot superfamily (Xenopus laevis)
1.557122764	Pcolce2	procollagen C-endopeptidase enhancer 2
1.557122764	Col3a1	procollagen, type III, alpha 1
1.557122764	Dpt	dermatopontin
1.557122764	Slc38a3	solute carrier family 38, member 3
1.626714843	Myohd1	myosin head domain containing 1
1.626714843	4931419k03Rik	RIKEN cDNA 4931419K03 gene
1.626714843	1500032d16Rik	RIKEN cDNA 1500032D16 gene
1.626714843	Cd99l2	Cd99 antigen-like 2
1.626714843	S100a14	S100 calcium binding protein A14
1.626714843	Ndufab1	NADH dehydrogenase (ubiquinone) 1, alpha/beta subcomplex, 1
1.626714843	Efemp2	epidermal growth factor-containing fibulin-like extracellular matrix protein 2
1.626714843	Cnih	cornichon homolog (Drosophila)
1.626714843	Smpd1	sphingomyelin phosphodiesterase 1, acid lysosomal
1.626714843	Sfxn1	sideroflexin 1
1.626714843	Tsen2	tRNA splicing endonuclease 2 homolog (SEN2, S. cerevisiae)
1.626714843	0610009o20Rik	RIKEN cDNA 0610009O20 gene
1.626714843	Prdx3	peroxiredoxin 3
1.626714843	2010107g23Rik	RIKEN cDNA 2010107G23 gene
1.626714843	1110004b13Rik	RIKEN cDNA 1110004B13 gene
1.626714843	Nek7	NIMA (never in mitosis gene a)-related expressed kinase 7
1.626714843	Scyl3	SCY1-like 3 (S. cerevisiae)
1.626714843	MGI:1925867	CSA-conditional, T cell activation-dependent protein
1.626714843	9330196j05Rik	RIKEN cDNA 9330196J05 gene
1.626714843	1810021j13Rik	RIKEN cDNA 1810021J13 gene
1.626714843	6230427j02Rik	RIKEN cDNA 6230427J02 gene
1.626714843	Clec11a	C-type lectin domain family 11, member a
1.626714843	Agtr1	angiotensin receptor 1
1.626714843	Dhrs7	dehydrogenase/reductase (SDR family) member 7
1.626714843	Sfxn4	sideroflexin 4
1.626714843	2010003o18Rik	RIKEN cDNA 2010003O18 gene
1.626714843	0610040b21Rik	RIKEN cDNA 0610040B21 gene
1.626714843	6430520m22Rik	RIKEN cDNA 6430520M22 gene
1.626714843	2010011i20Rik	RIKEN cDNA 2010011I20 gene
1.626714843	Ai604832	expressed sequence AI604832
1.626714843	Grb7	growth factor receptor bound protein 7
1.626714843	Anapc4	anaphase promoting complex subunit 4
1.626714843	Zfp322a	zinc finger protein 322a
1.626714843	Scd1	stearoyl-Coenzyme A desaturase 1
1.626714843	2610318k02Rik	RIKEN cDNA 2610318K02 gene
1.626714843	Iqcc	IQ motif containing C
1.626714843	Tmem17	transmembrane protein 17
1.626714843	Acad9	acyl-Coenzyme A dehydrogenase family, member 9
1.626714843	Mrpl36	mitochondrial ribosomal protein L36
1.626714843	Timp4	tissue inhibitor of metalloproteinase 4
1.626714843	Nif3l1	Ngg1 interacting factor 3-like 1 (S. pombe)
1.626714843	3110043j09Rik	RIKEN cDNA 3110043J09 gene
1.626714843	Epha7	Eph receptor A7
1.626714843	Zdhhc12	zinc finger, DHHC domain containing 12
1.626714843	Tirap	toll-interleukin 1 receptor (TIR) domain-containing adaptor protein
1.626714843	Fcmd	Fukuyama type congenital muscular dystrophy homolog (human)
1.626714843	Bc027828	cDNA sequence BC027828
1.626714843	Dnalc4	dynein, axonemal, light chain 4
1.626714843	Ly6a	lymphocyte antigen 6 complex, locus A
1.626714843	Wdr45	WD repeat domain 45
1.626714843	Tmed5	transmembrane emp24 protein transport domain containing 5
1.626714843	Znf23	zinc finger protein 23 (KOX 16)
1.626714843	Pttg1ip	pituitary tumor-transforming 1 interacting protein
1.626714843	Trim29	tripartite motif protein 29
1.626714843	C630013n10Rik	RIKEN cDNA C630013N10 gene
1.626714843	Fzd1	frizzled homolog 1 (Drosophila)
1.895021888	Aldh6a1	aldehyde dehydrogenase family 6, subfamily A1
1.895021888	Rohn	ras homolog gene family, member N
1.895021888	Ai842396	expressed sequence AI842396
1.895021888	Ndn	necdin
1.895021888	Ndufa4	NADH dehydrogenase (ubiquinone) 1 alpha subcomplex, 4
1.895021888	Rabl4	RAB, member of RAS oncogene family-like 4
1.895021888	Mr1	major histocompatibility complex, class I-related

1.895021888	Gemin4	gem (nuclear organelle) associated protein 4
1.895021888	Tnnt3	troponin T3, skeletal, fast
1.895021888	C2	complement component 2 (within H-2S)
1.895021888	Rfk	riboflavin kinase
1.895021888	Extl1	exostoses (multiple)-like 1
1.895021888	Cd36	CD36 antigen
1.895021888	6030490i01Rik	RIKEN cDNA 6030490I01 gene
1.895021888	1200009f10Rik	RIKEN cDNA 1200009F10 gene
1.895021888	Fbn1	fibrillin 1
1.895021888	C78409	expressed sequence C78409
1.895021888	Paqr6	progestin and adipoQ receptor family member VI
1.895021888	4932411a10Rik	RIKEN cDNA 4932411A10 gene
1.895021888	Ai662250	expressed sequence AI662250
1.895021888	Abcb6	ATP-binding cassette, sub-family B (MDR/TAP), member 6
1.895021888	Plscr3	phospholipid scramblase 3
1.895021888	Bckdk	branched chain ketoacid dehydrogenase kinase
1.895021888	Trim23	tripartite motif protein 23
1.895021888	Perp	PERP, TP53 apoptosis effector
1.895021888	MGI:1920480	BMP-binding endothelial regulator
1.895021888	1110058l19Rik	RIKEN cDNA 1110058L19 gene
1.895021888	Cbx2	chromobox homolog 2 (Drosophila Pc class)
1.895021888	Pros1	protein S (alpha)
1.895021888	Lamb2	laminin, beta 2
1.895021888	Pex11a	peroxisomal biogenesis factor 11a
1.895021888	Lxn	latexin
1.895021888	Zfp426	zinc finger protein 426
1.895021888	Tcfap2c	transcription factor AP-2, gamma
1.895021888	4933425f03Rik	RIKEN cDNA 4933425F03 gene
1.895021888	Wbscr16	Williams-Beuren syndrome chromosome region 16 homolog (human)
1.895021888	Dci	dodecenoyl-Coenzyme A delta isomerase (3,2 trans-enoyl-Coenyme A isomerase)
1.895021888	D12ertd647e	DNA segment, Chr 12, ERATO Doi 647, expressed
1.895021888	4930577m16Rik	RIKEN cDNA 4930577M16 gene
1.895021888	Cdw92	CDW92 antigen
1.895021888	Cd151	CD151 antigen
1.895021888	Scrg1	scrapie responsive gene 1
1.895021888	Cplx2	complexin 2
1.895021888	2410005o16Rik	RIKEN cDNA 2410005O16 gene
1.895021888	Slc25a29	solute carrier family 25 (mitochondrial carrier, palmitoylcarnitine transporter), member 29
1.895021888	Rnmtl1	RNA methyltransferase like 1
1.895021888	Cog8	component of oligomeric golgi complex 8
1.895021888	Plk2	polo-like kinase 2 (Drosophila)
1.895021888	Spcs1	signal peptidase complex subunit 1 homolog (S. cerevisiae)
1.895021888	4430402i18Rik	RIKEN cDNA 4430402I18 gene
1.895021888	Cab39l	calcium binding protein 39-like
1.895021888	Slc25a17	solute carrier family 25 (mitochondrial carrier, peroxisomal membrane protein), member 17
1.895021888	Fbp1	fructose bisphosphatase 1
1.895021888	2610311i19Rik	RIKEN cDNA 2610311I19 gene
1.895021888	Hdhd3	haloacid dehalogenase-like hydrolase domain containing 3
1.895021888	Cdc16	CDC16 cell division cycle 16 homolog (S. cerevisiae)
1.895021888	Aw146020	expressed sequence AW146020
1.895021888	Rhoc	ras homolog gene family, member C
1.895021888	Mmp2	matrix metalloproteinase 2
1.895021888	Rdh14	retinol dehydrogenase 14 (all-trans and 9-cis)
1.895021888	Jub	ajuba
1.895021888	Clpb	ClpB caseinolytic peptidase B homolog (E. coli)
1.895021888	Parp16	poly (ADP-ribose) polymerase family, member 16
1.895021888	Fabp9	fatty acid binding protein 9, testis
1.895021888	Ltbp3	latent transforming growth factor beta binding protein 3
1.895021888	1810033a06Rik	RIKEN cDNA 1810033A06 gene
1.895021888	Sema3c	sema domain, immunoglobulin domain (Ig), short basic domain, secreted, (semaphorin) 3C
1.895021888	2610205e22Rik	RIKEN cDNA 2610205E22 gene
1.895021888	Adcyap1r1	adenylate cyclase activating polypeptide 1 receptor 1
1.895021888	Mrps17	mitochondrial ribosomal protein S17
1.895021888	Zdhhc16	zinc finger, DHHC domain containing 16
1.895021888	D11ertd730e	DNA segment, Chr 11, ERATO Doi 730, expressed
1.895021888	1600012h06Rik	RIKEN cDNA 1600012H06 gene
1.895021888	0610012d09Rik	RIKEN cDNA 0610012D09 gene
1.895021888	MGI:1921571	timeless interacting protein
1.895021888	Dok4	docking protein 4
1.895021888	2610044o15Rik	RIKEN cDNA 2610044O15 gene
2.021069957	Nfatc4	nuclear factor of activated T-cells, cytoplasmic, calcineurin-dependent 4
2.021069957	2210021j22Rik	RIKEN cDNA 2210021J22 gene
2.021069957	Dph2	DPH2 homolog (S. cerevisiae)
2.021069957	Asah2	N-acylsphingosine amidohydrolase 2
2.021069957	Aig1	androgen-induced 1
2.021069957	MGI:2385656	lipidosin
2.021069957	A230106m15Rik	RIKEN cDNA A230106M15 gene
2.021069957	Nr5a2	nuclear receptor subfamily 5, group A, member 2
2.021069957	Krt1-19	keratin complex 1, acidic, gene 19
2.021069957	Atp5s	ATP synthase, H+ transporting, mitochondrial F0 complex, subunit s
2.021069957	Tspan6	tetraspanin 6
2.021069957	Tle6	transducin-like enhancer of split 6, homolog of Drosophila E(spl)
2.021069957	Col1a2	procollagen, type I, alpha 2
2.021069957	Rnf103	ring finger protein 103
2.021069957	Zfp105	zinc finger protein 105
2.021069957	2310039h08Rik	RIKEN cDNA 2310039H08 gene
2.021069957	Loxl1	lysyl oxidase-like 1

2.021069957	Cav2	caveolin 2
2.021069957	Thtpa	thiamine triphosphatase
2.021069957	4632417n05Rik	RIKEN cDNA 4632417N05 gene
2.021069957	Col5a1	procollagen, type V, alpha 1
2.021069957	Eps8l2	EPS8-like 2
2.021069957	2810432d09Rik	RIKEN cDNA 2810432D09 gene
2.021069957	Cecr5	cat eye syndrome chromosome region, candidate 5 homolog (human)
2.021069957	Tmem18	transmembrane protein 18
2.021069957	Id4	inhibitor of DNA binding 4
2.021069957	Rabl3	RAB, member of RAS oncogene family-like 3
2.021069957	Wig1	wild-type p53-induced gene 1
2.021069957	Serf1a	small EDRK-rich factor 1A (telomeric)
2.021069957	Pbp	phosphatidylethanolamine binding protein
2.021069957	Osgepl1	O-sialoglycoprotein endopeptidase-like 1
2.021069957	Fgd1	FYVE, RhoGEF and PH domain containing 1
2.021069957	Dpagt1	dolichyl-phosphate (UDP-N-acetylglucosamine) acetylglucosaminephosphotransferase 1 (GlcNAc-1-P transferase)
2.021069957	Timm22	translocase of inner mitochondrial membrane 22 homolog (yeast)
2.021069957	Fbxo31	F-box only protein 31
2.021069957	Aa409316	expressed sequence AA409316
2.021069957	Nr2c1	nuclear receptor subfamily 2, group C, member 1
2.021069957	C76566	expressed sequence C76566
2.021069957	Hebp2	heme binding protein 2
2.021069957	Scrn3	secernin 3
2.021069957	MGI:1913996	dynein 2 light intermediate chain
2.021069957	Fcna	ficolin A
2.021069957	Prlr	prolactin receptor
2.021069957	Cdo1	cysteine dioxygenase 1, cytosolic
2.021069957	Muc1	mucin 1, transmembrane
2.021069957	Gja5	gap junction membrane channel protein alpha 5
2.021069957	1700018o18Rik	RIKEN cDNA 1700018O18 gene
2.021069957	1810060d16Rik	RIKEN cDNA 1810060D16 gene
2.021069957	Zfyve20	zinc finger, FYVE domain containing 20
2.021069957	1110059e24Rik	RIKEN cDNA 1110059E24 gene
2.021069957	2210010b09Rik	RIKEN cDNA 2210010B09 gene
2.021069957	Aldh5a1	aldehyde dehydrogenase family 5, subfamily A1
2.021069957	Ankmy2	ankyrin repeat and MYND domain containing 2
2.021069957	C1r	complement component 1, r subcomponent
2.021069957	1810049h13Rik	RIKEN cDNA 1810049H13 gene
2.021069957	4632433k11Rik	RIKEN cDNA 4632433K11 gene
2.021069957	1810043g02Rik	RIKEN cDNA 1810043G02 gene
2.021069957	Cops5	COP9 (constitutive photomorphogenic) homolog, subunit 5 (Arabidopsis thaliana)
2.021069957	Zfp39	zinc finger protein 39
2.021069957	Bet1	blocked early in transport 1 homolog (S. cerevisiae)
2.021069957	D8ertd812e	DNA segment, Chr 8, ERATO Doi 812, expressed
2.021069957	Wtip	WT1-interacting protein
2.021069957	Itm2a	integral membrane protein 2A
2.021069957	Parn	poly(A)-specific ribonuclease (deadenylation nuclease)
2.021069957	Bc026778	cDNA sequence BC026778
2.021069957	B230317c12Rik	RIKEN cDNA B230317C12 gene
2.021069957	Tlr3	toll-like receptor 3
2.021069957	Fmod	fibromodulin
2.021069957	Fzd7	frizzled homolog 7 (Drosophila)
2.021069957	Osbpl2	oxysterol binding protein-like 2
2.021069957	Nit1	nitrilase 1
2.021069957	Decr1	2,4-dienoyl CoA reductase 1, mitochondrial
2.021069957	Bc052066	cDNA sequence BC052066
2.021069957	3110037i16Rik	RIKEN cDNA 3110037I16 gene
2.021069957	Ptk2	PTK2 protein tyrosine kinase 2
2.021069957	Nme3	expressed in non-metastatic cells 3
2.021069957	Smarcd3	SWI/SNF related, matrix associated, actin dependent regulator of chromatin, subfamily d, member 3
2.021069957	Map1lc3b	microtubule-associated protein 1 light chain 3 beta
2.021069957	Dmap1	DNA methyltransferase 1-associated protein 1
2.021069957	Urod	uroporphyrinogen decarboxylase
2.021069957	Prrg2	proline-rich Gla (G-carboxyglutamic acid) polypeptide 2
2.021069957	D10ertd610e	DNA segment, Chr 10, ERATO Doi 610, expressed
2.021069957	Nol7	nucleolar protein 7
2.021069957	D4bwg0951e	DNA segment, Chr 4, Brigham & Women's Genetics 0951 expressed
2.021069957	Ndufc1	NADH dehydrogenase (ubiquinone) 1, subcomplex unknown, 1
2.021069957	Akr1b8	aldo-keto reductase family 1, member B8
2.021069957	Msrb2	methionine sulfoxide reductase B2
2.021069957	Abi2	abl-interactor 2
2.021069957	Ai597479	expressed sequence AI597479
2.021069957	Gstt3	glutathione S-transferase, theta 3
2.021069957	Col5a3	procollagen, type V, alpha 3
2.021069957	Pecr	peroxisomal trans-2-enoyl-CoA reductase
2.188047709	Egfr	epidermal growth factor receptor
2.188047709	Znhit2	zinc finger, HIT domain containing 2
2.188047709	Rarres2	retinoic acid receptor responder (tazarotene induced) 2
2.188047709	Olfml2b	olfactomedin-like 2B
2.188047709	1810014f10Rik	RIKEN cDNA 1810014F10 gene
2.188047709	Ggcx	gamma-glutamyl carboxylase
2.188047709	Bc016198	cDNA sequence BC016198
2.188047709	6530411b15Rik	RIKEN cDNA 6530411B15 gene
2.188047709	Galk1	galactokinase 1
2.188047709	Bc006662	cDNA sequence BC006662
2.188047709	Nipa1	non imprinted in Prader-Willi/Angelman syndrome 1 homolog (human)
2.188047709	A230050p20Rik	RIKEN cDNA A230050P20 gene

2.188047709	Cldn10	claudin 10
2.188047709	4432406c05Rik	RIKEN cDNA 4432406C05 gene
2.188047709	Biklk	Bcl2-interacting killer-like
2.188047709	Tparl	TPA regulated locus
2.188047709	1700039d13Rik	RIKEN cDNA 1700039D13 gene
2.188047709	Bag4	BCL2-associated athanogene 4
2.188047709	9330164h19Rik	RIKEN cDNA 9330164H19 gene
2.188047709	Acy3	aspartoacylase (aminoacylase) 3
2.188047709	Serpina3n	serine (or cysteine) proteinase inhibitor, clade A, member 3N
2.188047709	Slc25a4	solute carrier family 25 (mitochondrial carrier, adenine nucleotide translocator), member 4
2.188047709	Hrsp12	heat-responsive protein 12
2.188047709	Rcn1	reticulocalbin 1
2.188047709	Nme7	non-metastatic cells 7, protein expressed in
2.188047709	Chchd1	coiled-coil-helix-coiled-coil-helix domain containing 1
2.188047709	Aass	aminoadipate-semialdehyde synthase
2.188047709	Aw742319	expressed sequence AW742319
2.188047709	Ednrb	endothelin receptor type B
2.188047709	Cpt1c	carnitine palmitoyltransferase 1c
2.188047709	Pex11c	peroxisomal biogenesis factor 11c
2.188047709	5031439a09Rik	RIKEN cDNA 5031439A09 gene
2.188047709	Agtrl1	angiotensin receptor-like 1
2.188047709	Elovl7	ELOVL family member 7, elongation of long chain fatty acids (yeast)
2.188047709	Ccl24	chemokine (C-C motif) ligand 24
2.188047709	0610006i08Rik	RIKEN cDNA 0610006I08 gene
2.188047709	Tmeff2	transmembrane protein with EGF-like and two follistatin-like domains 2
2.188047709	Bc010552	cDNA sequence BC010552
2.188047709	4931406c07Rik	RIKEN cDNA 4931406C07 gene
2.188047709	2610208e05Rik	RIKEN cDNA 2610208E05 gene
2.188047709	2410011g03Rik	RIKEN cDNA 2410011G03 gene
2.188047709	Irx5	Iroquois related homeobox 5 (Drosophila)
2.188047709	Rnf3	ring finger protein 3
2.188047709	Aw060207	expressed sequence AW060207
2.188047709	Gng12	guanine nucleotide binding protein (G protein), gamma 12
2.188047709	Lama4	laminin, alpha 4
2.188047709	Crip2	cysteine rich protein 2
2.188047709	Tfdp1	transcription factor Dp 1
2.188047709	Tnnc2	troponin C2, fast
2.324098412	Elmo3	engulfment and cell motility 3, ced-12 homolog (C. elegans)
2.324098412	1810009b06Rik	RIKEN cDNA 1810009B06 gene
2.324098412	Gas1	growth arrest specific 1
2.324098412	Maged1	melanoma antigen, family D, 1
2.324098412	2700055k07Rik	RIKEN cDNA 2700055K07 gene
2.324098412	Hic1	hypermethylated in cancer 1
2.324098412	Aoc3	amine oxidase, copper containing 3
2.324098412	Bag2	Bcl2-associated athanogene 2
2.324098412	B430320c24Rik	RIKEN cDNA B430320C24 gene
2.324098412	Fem1a	feminization 1 homolog a (C. elegans)
2.324098412	4933428g09Rik	RIKEN cDNA 4933428G09 gene
2.324098412	Osr2	odd-skipped related 2 (Drosophila)
2.324098412	Serpinh1	serine (or cysteine) proteinase inhibitor, clade H, member 1
2.324098412	Glg1	golgi apparatus protein 1
2.324098412	2310067g05Rik	RIKEN cDNA 2310067G05 gene
2.324098412	Surf1	surfeit gene 1
2.324098412	6820429m01	hypothetical protein 6820429M01
2.324098412	Hibadh	3-hydroxyisobutyrate dehydrogenase
2.324098412	Zfp503	zinc finger protein 503
2.324098412	Pgam2	phosphoglycerate mutase 2
2.324098412	Scn1b	sodium channel, voltage-gated, type I, beta
2.324098412	Bc038925	cDNA sequence BC038925
2.324098412	Pja2	praja 2, RING-H2 motif containing
2.324098412	Zdhhc1	zinc finger, DHHC domain containing 1
2.324098412	Wbscr18	Williams-Beuren syndrome chromosome region 18 homolog (human)
2.324098412	Nfe2l3	nuclear factor, erythroid derived 2, like 3
2.324098412	Fzd6	frizzled homolog 6 (Drosophila)
2.324098412	Spg20	spastic paraplegia 20, spartin (Troyer syndrome) homolog (human)
2.324098412	Ecm1	extracellular matrix protein 1
2.324098412	1110001c20Rik	RIKEN cDNA 1110001C20 gene
2.324098412	9030425e11Rik	RIKEN cDNA 9030425E11 gene
2.324098412	6720467c03Rik	RIKEN cDNA 6720467C03 gene
2.324098412	Armc1	armadillo repeat containing 1
2.324098412	Chgb	chromogranin B
2.324098412	4632406n01Rik	RIKEN cDNA 4632406N01 gene
2.324098412	Av249152	expressed sequence AV249152
2.324098412	2310005o14Rik	RIKEN cDNA 2310005O14 gene
2.324098412	Abhd4	abhydrolase domain containing 4
2.324098412	Mmaa	methylmalonic aciduria (cobalamin deficiency) type A
2.324098412	Irf6	interferon regulatory factor 6
2.324098412	Gpd1	glycerol-3-phosphate dehydrogenase 1 (soluble)
2.324098412	Mpv17	Mpv17 transgene, kidney disease mutant
2.324098412	Mesdc2	mesoderm development candiate 2
2.324098412	Sdc2	syndecan 2
2.324098412	Zcchc3	zinc finger, CCHC domain containing 3
2.324098412	Cluap1	clusterin associated protein 1
2.324098412	MGI:2179725	candidate tumor suppressor OVCA2
2.324098412	Med19	mediator of RNA polymerase II transcription, subunit 19 homolog (yeast)
2.324098412	Slc2a10	solute carrier family 2 (facilitated glucose transporter), member 10
2.324098412	Cmkor1	chemokine orphan receptor 1

2.324098412	2310016c16Rik	RIKEN cDNA 2310016C16 gene
2.324098412	Glt8d1	glycosyltransferase 8 domain containing 1
2.324098412	Ehhadh	enoyl-Coenzyme A, hydratase/3-hydroxyacyl Coenzyme A dehydrogenase
2.324098412	Gstt2	glutathione S-transferase, theta 2
2.324098412	4831416g18Rik	RIKEN cDNA 4831416G18 gene
2.324098412	9030611o19Rik	RIKEN cDNA 9030611O19 gene
2.324098412	Hoxc9	homeo box C9
2.324098412	Ttc8	tetratricopeptide repeat domain 8
2.324098412	Rundc1	RUN domain containing 1
2.324098412	Hey1	hairy/enhancer-of-split related with YRPW motif 1
2.324098412	Poldip2	polymerase (DNA-directed), delta interacting protein 2
2.324098412	Cul7	cullin 7
2.324098412	Carkl	carbohydrate kinase-like
2.324098412	Rbp7	retinol binding protein 7, cellular
2.324098412	Cox8b	cytochrome c oxidase, subunit VIIIb
2.324098412	8030466o12Rik	RIKEN cDNA 8030466O12 gene
2.324098412	Nap1l3	nucleosome assembly protein 1-like 3
2.324098412	Pcgf6	polycomb group ring finger 6
2.324098412	Cant1	calcium activated nucleotidase 1
2.324098412	Rhob	ras homolog gene family, member B
2.324098412	Cfl2	cofilin 2, muscle
2.324098412	Chst8	carbohydrate (N-acetylgalactosamine 4-0) sulfotransferase 8
2.324098412	5730409k12Rik	RIKEN cDNA 5730409K12 gene
2.324098412	Sco1	SCO cytochrome oxidase deficient homolog 1 (yeast)
2.324098412	1110007f12Rik	RIKEN cDNA 1110007F12 gene
2.324098412	Wdr35	WD repeat domain 35
2.324098412	Fkbp7	FK506 binding protein 7
2.324098412	Denr	density-regulated protein
2.324098412	Grinl1a	glutamate receptor, ionotropic, N-methyl D-aspartate-like 1A
2.324098412	Bc005471	cDNA sequence BC005471
2.324098412	Gtf2h3	general transcription factor IIH, polypeptide 3
2.324098412	MGI:1889209	semaF cytoplasmic domain associated protein 2
2.324098412	Igfbp5	insulin-like growth factor binding protein 5
2.324098412	Gpt2	glutamic pyruvate transaminase (alanine aminotransferase) 2
2.433004319	Spnb3	spectrin beta 3
2.433004319	Pdcd2	programmed cell death 2
2.433004319	Kdelr1	KDEL (Lys-Asp-Glu-Leu) endoplasmic reticulum protein retention receptor 1
2.433004319	2610319k07Rik	RIKEN cDNA 2610319K07 gene
2.433004319	Timp2	tissue inhibitor of metalloproteinase 2
2.433004319	C530028i08Rik	RIKEN cDNA C530028I08 gene
2.433004319	Cys1	cystin 1
2.433004319	Maob	monoamine oxidase B
2.433004319	4930403j22Rik	RIKEN cDNA 4930403J22 gene
2.433004319	2610008e11Rik	RIKEN cDNA 2610008E11 gene
2.433004319	Ddx28	DEAD (Asp-Glu-Ala-Asp) box polypeptide 28
2.433004319	Dbt	dihydrolipoamide branched chain transacylase E2
2.433004319	D930005d10Rik	RIKEN cDNA D930005D10 gene
2.433004319	Figf	c-fos induced growth factor
2.433004319	Ndufs1	NADH dehydrogenase (ubiquinone) Fe-S protein 1
2.433004319	Nrbp2	nuclear receptor binding protein 2
2.433004319	5430407p10Rik	RIKEN cDNA 5430407P10 gene
2.433004319	Fundc2	FUN14 domain containing 2
2.433004319	Sall2	sal-like 2 (Drosophila)
2.433004319	Ccdc5	coiled-coil domain containing 5
2.433004319	1190005i06Rik	RIKEN cDNA 1190005I06 gene
2.433004319	Ly6d	lymphocyte antigen 6 complex, locus D
2.433004319	Ryk	receptor-like tyrosine kinase
2.433004319	Tbx18	T-box18
2.433004319	Bc022687	cDNA sequence BC022687
2.433004319	Spon2	spondin 2, extracellular matrix protein
2.433004319	Tpi1	triosephosphate isomerase 1
2.433004319	Eif2b1	eukaryotic translation initiation factor 2B, subunit 1 (alpha)
2.433004319	Rwdd3	RWD domain containing 3
2.433004319	Insig2	insulin induced gene 2
2.433004319	Acadsb	acyl-Coenzyme A dehydrogenase, short/branched chain
2.433004319	Irx2	Iroquois related homeobox 2 (Drosophila)
2.433004319	Zfp526	zinc finger protein 526
2.433004319	Chga	chromogranin A
2.433004319	Zfp354a	zinc finger protein 354A
2.433004319	Mrpl22	mitochondrial ribosomal protein L22
2.433004319	Pfdn1	prefoldin 1
2.433004319	Sh3tc2	SH3 domain and tetratricopeptide repeats 2
2.433004319	Khdrbs3	KH domain containing, RNA binding, signal transduction associated 3
2.433004319	Bcl2l14	Bcl2-like 14 (apoptosis facilitator)
2.433004319	1700028n11Rik	RIKEN cDNA 1700028N11 gene
2.433004319	Jund1	Jun proto-oncogene related gene d1
2.433004319	5730568a12Rik	RIKEN cDNA 5730568A12 gene
2.433004319	Smyd2	SET and MYND domain containing 2
2.433004319	Nfs1	nitrogen fixation gene 1 (S. cerevisiae)
2.433004319	Tspan1	tetraspan 1
2.433004319	Ptk9	protein tyrosine kinase 9
2.433004319	Timm8b	translocase of inner mitochondrial membrane 8 homolog b (yeast)
2.433004319	MGI:105968	taxilin
2.433004319	1700108j01Rik	RIKEN cDNA 1700108J01 gene
2.433004319	6530401n04Rik	RIKEN cDNA 6530401N04 gene
2.433004319	D11ertd333e	DNA segment, Chr 11, ERATO Doi 333, expressed
2.433004319	Rasgrp3	RAS, guanyl releasing protein 3

2.433004319	Zcchc10	zinc finger, CCHC domain containing 10
2.433004319	H1f0	H1 histone family, member 0
2.433004319	Hcfc1r1	host cell factor C1 regulator 1 (XPO1-dependent)
2.433004319	Nisch	nischarin
2.433004319	Rhod	ras homolog gene family, member D
2.433004319	6430601a21Rik	RIKEN cDNA 6430601A21 gene
2.433004319	Apbb1	amyloid beta (A4) precursor protein-binding, family B, member 1
2.433004319	Fkbp9	FK506 binding protein 9
2.433004319	Atpif1	ATPase inhibitory factor 1
2.433004319	Actr6	ARP6 actin-related protein 6 homolog (yeast)
2.433004319	5730466h23Rik	RIKEN cDNA 5730466H23 gene
2.433004319	Atp1b1	ATPase, Na+/K+ transporting, beta 1 polypeptide
2.433004319	Eln	elastin
2.433004319	Ai481750	expressed sequence AI481750
2.433004319	Pcdhb22	protocadherin beta 22
2.433004319	Pdgfra	platelet derived growth factor receptor, alpha polypeptide
2.433004319	9030405d14Rik	RIKEN cDNA 9030405D14 gene
2.433004319	Icrfp703b1614q5.6	predicted gene ICRFP703B1614Q5.6
2.433004319	Dag1	dystroglycan 1
2.433004319	2400006a19Rik	RIKEN cDNA 2400006A19 gene
2.433004319	Atf7	activating transcription factor 7
2.433004319	Pex6	peroxisomal biogenesis factor 6
2.433004319	E430012m05Rik	RIKEN cDNA E430012M05 gene
2.433004319	Ccnj	cyclin J
2.433004319	Nudt6	nudix (nucleoside diphosphate linked moiety X)-type motif 6
2.433004319	Dpep1	dipeptidase 1 (renal)
2.433004319	Dapk3	death-associated kinase 3
2.433004319	Gpatc1	G patch domain containing 1
2.433004319	Zfp482	zinc finger protein 482
2.433004319	Tbcc	tubulin-specific chaperone c
2.433004319	Gas6	growth arrest specific 6
2.433004319	Amacr	alpha-methylacyl-CoA racemase
2.433004319	2810003c17Rik	RIKEN cDNA 2810003C17 gene
2.433004319	2310066i18Rik	RIKEN cDNA 2310066I18 gene
2.433004319	MGI:2678023	homeodomain leucine zipper-encoding gene
2.433004319	Sult5a1	sulfotransferase family 5A, member 1
2.433004319	Krt2-7	keratin complex 2, basic, gene 7
2.433004319	Gprc5c	G protein-coupled receptor, family C, group 5, member C
2.433004319	Smpd3	sphingomyelin phosphodiesterase 3, neutral
2.433004319	Gkap1	G kinase anchoring protein 1
2.433004319	Acadl	acetyl-Coenzyme A dehydrogenase, long-chain
2.433004319	Clpx	caseinolytic protease X (E.coli)
2.433004319	Slc7a2	solute carrier family 7 (cationic amino acid transporter, y+ system), member 2
2.433004319	Lu	Lutheran blood group (Auberger b antigen included)
2.433004319	C330018k18Rik	RIKEN cDNA C330018K18 gene
2.433004319	1810037c20Rik	RIKEN cDNA 1810037C20 gene
2.433004319	Rnf144	ring finger protein 144
2.433004319	Lace1	lactation elevated 1
2.433004319	9830169c18Rik	RIKEN cDNA 9830169C18 gene
2.433004319	Igfbp4	insulin-like growth factor binding protein 4
2.433004319	Ebp	phenylalkylamine Ca2+ antagonist (emopamil) binding protein
2.433004319	1810011o10Rik	RIKEN cDNA 1810011O10 gene
2.433004319	Acads	acyl-Coenzyme A dehydrogenase, short chain
2.433004319	D830019k17Rik	RIKEN cDNA D830019K17 gene
2.433004319	2610317d23Rik	RIKEN cDNA 2610317D23 gene
2.433004319	Pgea1	PKD2 interactor, golgi and endoplasmic reticulum associated 1
2.433004319	Slc30a6	solute carrier family 30 (zinc transporter), member 6
2.433004319	1700006d24Rik	RIKEN cDNA 1700006D24 gene
2.433004319	2900070e19Rik	RIKEN cDNA 2900070E19 gene
2.433004319	8430427h17Rik	RIKEN cDNA 8430427H17 gene
2.433004319	Creld1	cysteine-rich with EGF-like domains 1
2.433004319	MGI:2136405	glucuronyl C5-epimerase
2.433004319	Rab18	RAB18, member RAS oncogene family
2.433004319	2700097o09Rik	RIKEN cDNA 2700097O09 gene
2.433004319	Podxl	podocalyxin-like
2.433004319	Bbs4	Bardet-Biedl syndrome 4 homolog (human)
2.433004319	1810042k04Rik	RIKEN cDNA 1810042K04 gene
2.433004319	0610006o14Rik	RIKEN cDNA 0610006O14 gene
2.433004319	Stx17	syntaxin 17
2.433004319	Klf2	Kruppel-like factor 2 (lung)
2.433004319	Lgals7	lectin, galactose binding, soluble 7
2.433004319	Rassf6	Ras association (RalGDS/AF-6) domain family 6
2.433004319	Nbl1	neuroblastoma, suppression of tumorigenicity 1
2.433004319	Gpr89	G protein-coupled receptor 89
2.433004319	Xpc	xeroderma pigmentosum, complementation group C
2.433004319	Ndufb10	NADH dehydrogenase (ubiquinone) 1 beta subcomplex, 10
2.433004319	Cyp2d26	cytochrome P450, family 2, subfamily d, polypeptide 26
2.433004319	2310047K21Rik	RIKEN cDNA 2310047K21 gene
2.433004319	Bc018242	cDNA sequence BC018242
2.433004319	Itih2	inter-alpha trypsin inhibitor, heavy chain 2
2.433004319	Itgb6	integrin beta 6
2.799303861	Egr1	early growth response 1
2.799303861	Plek2	pleckstrin 2
2.799303861	Krt1-18	keratin complex 1, acidic, gene 18
2.799303861	Prkcz	protein kinase C, zeta
2.799303861	4921504n20Rik	RIKEN cDNA 4921504N20 gene
2.799303861	Ssr2	signal sequence receptor, beta

2.799303861	Parva	parvin, alpha
2.799303861	Polr3k	polymerase (RNA) III (DNA directed) polypeptide K
2.799303861	Fgfr2	fibroblast growth factor receptor 2
2.799303861	3000004n20Rik	RIKEN cDNA 3000004N20 gene
2.799303861	Elof1	elongation factor 1 homolog (ELF1, S. cerevisiae)
2.799303861	Acrbp	proacrosin binding protein
2.799303861	St14	suppression of tumorigenicity 14 (colon carcinoma)
2.799303861	Suclg1	succinate-CoA ligase, GDP-forming, alpha subunit
2.799303861	Nudt12	nudix (nucleoside diphosphate linked moiety X)-type motif 12
2.799303861	Rnf13	ring finger protein 13
2.799303861	Pex19	peroxisome biogenesis factor 19
2.799303861	Poll	polymerase (DNA directed), lambda
2.799303861	Znf250	zinc finger protein 250
2.799303861	Mrps22	mitochondrial ribosomal protein S22
2.799303861	Bc006705	cDNA sequence BC006705
2.799303861	Mfn2	mitofusin 2
2.799303861	Wdr39	WD repeat domain 39
2.799303861	Lgr6	leucine-rich repeat-containing G protein-coupled receptor 6
2.799303861	9630054f20Rik	RIKEN cDNA 9630054F20 gene
2.799303861	Gm944	gene model 944, (NCBI)
2.799303861	Krt2-6b	keratin complex 2, basic, gene 6b
2.799303861	Bc034099	cDNA sequence BC034099
2.799303861	Nr1i4	nuclear receptor subfamily 1, group I, member 4
2.799303861	Acadm	acetyl-Coenzyme A dehydrogenase, medium chain
2.799303861	Slc2a4	solute carrier family 2 (facilitated glucose transporter), member 4
2.799303861	Zfp68	zinc finger protein 68
2.799303861	Vps45a	vacuolar protein sorting 45A (yeast)
2.799303861	E430034l04Rik	RIKEN cDNA E430034L04 gene
2.799303861	Idh3a	isocitrate dehydrogenase 3 (NAD+) alpha
2.799303861	Epm2aip1	EPM2A (laforin) interacting protein 1
2.799303861	Nutf2	nuclear transport factor 2
2.799303861	Pfkm	phosphofructokinase, muscle
2.799303861	Tmem14a	transmembrane protein 14A
2.799303861	Des	desmin
2.799303861	Ptov1	prostate tumor over expressed gene 1
2.799303861	Yif1	Yip1 interacting factor homolog (S. cerevisiae)
2.799303861	Mrps35	mitochondrial ribosomal protein S35
2.799303861	Narg2	NMDA receptor-regulated gene 2
2.799303861	Bc024063	cDNA sequence BC024063
2.799303861	Baiap2l1	BAI1-associated protein 2-like 1
2.799303861	Zfp75	zinc finger protein 75
2.799303861	Polrmt	polymerase (RNA) mitochondrial (DNA directed)
2.799303861	4732418c07Rik	RIKEN cDNA 4732418C07 gene
2.799303861	Usp20	ubiquitin specific protease 20
2.799303861	Prkaa2	protein kinase, AMP-activated, alpha 2 catalytic subunit
2.799303861	1500041b16Rik	RIKEN cDNA 1500041B16 gene
2.799303861	Snrk	SNF related kinase
2.799303861	Wdr21	WD repeat domain 21
2.799303861	Cd81	CD 81 antigen
2.799303861	Lifr	leukemia inhibitory factor receptor
2.799303861	Cul4a	cullin 4A
2.799303861	3110048e14Rik	RIKEN cDNA 3110048E14 gene
2.799303861	Smtn	smoothelin
2.799303861	Slc25a24	solute carrier family 25 (mitochondrial carrier, phosphate carrier), member 24
2.799303861	Bc016226	cDNA sequence BC016226
2.799303861	3110050n22Rik	RIKEN cDNA 3110050N22 gene
2.799303861	Pycr2	pyrroline-5-carboxylate reductase family, member 2
2.799303861	Aw743872	expressed sequence AW743872
2.799303861	2610019p18Rik	RIKEN cDNA 2610019P18 gene
2.799303861	Trmt1	tRNA (5-methylaminomethyl-2-thiouridylate)-methyltransferase 1
2.799303861	MGI:2384914	Dip3 beta
2.799303861	Zfp623	zinc finger protein 623
2.799303861	B230118h07Rik	RIKEN cDNA B230118H07 gene
2.799303861	2410015m20Rik	RIKEN cDNA 2410015M20 gene
2.799303861	Zfp61	zinc finger protein 61
2.799303861	Vsig2	V-set and immunoglobulin domain containing 2
2.799303861	Nkiras1	NFKB inhibitor interacting Ras-like protein 1
2.799303861	Slc35c1	solute carrier family 35, member C1
2.799303861	Sfrp2	secreted frizzled-related sequence protein 2
2.799303861	Rer1	RER1 retention in endoplasmic reticulum 1 homolog (S. cerevisiae)
2.799303861	8230402k04Rik	RIKEN cDNA 8230402K04 gene
2.799303861	6330512m04Rik	RIKEN cDNA 6330512M04 gene
2.799303861	Sema4g	sema domain, immunoglobulin domain (Ig), transmembrane domain (TM) and short cytoplasmic domain, (semaphorin) 4G
2.799303861	Twsg1	twisted gastrulation homolog 1 (Drosophila)
2.799303861	Rasl12	RAS-like, family 12
2.799303861	1810063b05Rik	RIKEN cDNA 1810063B05 gene
2.799303861	Fads3	fatty acid desaturase 3
2.799303861	4930507c10Rik	RIKEN cDNA 4930507C10 gene
2.799303861	Ntn4	netrin 4
2.799303861	Keap1	kelch-like ECH-associated protein 1
2.799303861	Gnpat	glyceronephosphate O-acyltransferase
2.799303861	Fbxo9	f-box only protein 9
2.799303861	Arhgap5	Rho GTPase activating protein 5
2.799303861	Btbd6	BTB (POZ) domain containing 6
2.799303861	Bet1l	blocked early in transport 1 homolog (S. cerevisiae)-like
2.799303861	Scara3	scavenger receptor class A, member 3
2.799303861	2310022m17Rik	RIKEN cDNA 2310022M17 gene

2.799303861	Efnb1	ephrin B1
2.799303861	Colec11	collectin sub-family member 11
2.799303861	Rhobtb3	Rho-related BTB domain containing 3
2.799303861	3110056o03Rik	RIKEN cDNA 3110056O03 gene
2.799303861	2810422b04Rik	RIKEN cDNA 2810422B04 gene
2.799303861	4932442k08Rik	RIKEN cDNA 4932442K08 gene
2.799303861	Lrig3	leucine-rich repeats and immunoglobulin-like domains 3
2.799303861	Tcte1l	t-complex-associated-testis-expressed 1-like
2.799303861	Sorbs3	sorbin and SH3 domain containing 3
2.799303861	Rshl2	radial spokehead-like 2
2.799303861	Acta1	actin, alpha 1, skeletal muscle
2.799303861	Oplah	5-oxoprolinase (ATP-hydrolysing)
2.799303861	1810046j19Rik	RIKEN cDNA 1810046J19 gene
2.799303861	5430431g03Rik	RIKEN cDNA 5430431G03 gene
2.799303861	Guk1	guanylate kinase 1
2.799303861	Cdipt	CDP-diacylglycerol--inositol 3-phosphatidyltransferase (phosphatidylinositol synthase)
2.799303861	2610209m04Rik	RIKEN cDNA 2610209M04 gene
2.799303861	3110023e09Rik	RIKEN cDNA 3110023E09 gene
2.799303861	0610040j01Rik	RIKEN cDNA 0610040J01 gene
2.799303861	Mtap	methylthioadenosine phosphorylase
2.799303861	Brd3	bromodomain containing 3
2.799303861	Fbxo18	F-box protein 18
3.425670081	Scamp1	secretory carrier membrane protein 1
3.425670081	Fyco1	FYVE and coiled-coil domain containing 1
3.425670081	Mkrn2	makorin, ring finger protein, 2
3.425670081	Adh1a	alcohol dehydrogenase 1A (class I), alpha polypeptide
3.425670081	Polr2d	polymerase (RNA) II (DNA directed) polypeptide D
3.425670081	Dexi	dexamethasone-induced transcript
3.425670081	Erbb2	v-erb-b2 erythroblastic leukemia viral oncogene homolog 2, neuro/glioblastoma derived oncogene homolog (avian)
3.425670081	1110001a12Rik	RIKEN cDNA 1110001A12 gene
3.425670081	Cox7b	cytochrome c oxidase subunit VIIb
3.425670081	Aa986860	expressed sequence AA986860
3.425670081	Mycbp	c-myc binding protein
3.425670081	Tmem42	transmembrane protein 42
3.425670081	Areg	amphiregulin
3.425670081	E130306m17Rik	RIKEN cDNA E130306M17 gene
3.425670081	Pgrmc1	progesterone receptor membrane component 1
3.425670081	Casp7	caspase 7
3.425670081	Rnf14	ring finger protein 14
3.425670081	Skb1	SKB1 homolog (S. pombe)
3.425670081	1500031m22Rik	RIKEN cDNA 1500031M22 gene
3.425670081	0610039a15Rik	RIKEN cDNA 0610039A15 gene
3.425670081	Col6a2	procollagen, type VI, alpha 2
3.425670081	Tcta	T-cell leukemia translocation altered gene
3.425670081	Col4a1	procollagen, type IV, alpha 1
3.425670081	Araf	v-raf murine sarcoma 3611 viral oncogene homolog
3.425670081	5730521e12Rik	RIKEN cDNA 5730521E12 gene
3.425670081	Cirbp	cold inducible RNA binding protein
3.425670081	Pcgf4	polycomb group ring finger 4
3.425670081	Mesp2	mesoderm posterior 2
3.425670081	Ddah2	dimethylarginine dimethylaminohydrolase 2
3.425670081	Azi2	5-azacytidine induced gene 2
3.425670081	Loh12cr1	loss of heterozygosity, 12, chromosomal region 1 homolog (human)
3.425670081	1110032e23Rik	RIKEN cDNA 1110032E23 gene
3.425670081	Actn4	actinin alpha 4
3.425670081	Uchl4	ubiquitin carboxyl-terminal esterase L4
3.425670081	9130213b05Rik	RIKEN cDNA 9130213B05 gene
3.425670081	Dusp6	dual specificity phosphatase 6
3.425670081	Gosr1	golgi SNAP receptor complex member 1
3.425670081	0610037h22Rik	RIKEN cDNA 0610037H22 gene
3.425670081	Pdgfrl	platelet-derived growth factor receptor-like
3.425670081	Phyh	phytanoyl-CoA hydroxylase
3.425670081	Cxxc5	CXXC finger 5
3.425670081	6430596g11Rik	RIKEN cDNA 6430596G11 gene
3.425670081	Bc017158	cDNA sequence BC017158
3.425670081	D1bwg0212e	DNA segment, Chr 1, Brigham & Women's Genetics 0212 expressed
3.425670081	1810045k17Rik	RIKEN cDNA 1810045K17 gene
3.425670081	Abcb9	ATP-binding cassette, sub-family B (MDR/TAP), member 9
3.425670081	1110001a23Rik	RIKEN cDNA 1110001A23 gene
3.425670081	Rnpepl1	arginyl aminopeptidase (aminopeptidase B)-like 1
3.425670081	Nid2	nidogen 2
3.425670081	Pdlim3	PDZ and LIM domain 3
3.425670081	Them2	thioesterase superfamily member 2
3.425670081	Zfp212	Zinc finger protein 212
3.425670081	1110030h02Rik	RIKEN cDNA 1110030H02 gene
3.425670081	1200007b05Rik	RIKEN cDNA 1200007B05 gene
3.425670081	C80913	expressed sequence C80913
3.425670081	Scnn1a	sodium channel, nonvoltage-gated, type I, alpha
3.425670081	Loc383229	similar to hemoglobin alpha chain - slender loris
3.425670081	Padi3	peptidyl arginine deiminase, type III
3.425670081	D11ertd99e	DNA segment, Chr 11, ERATO Doi 99, expressed
3.425670081	Timm10	translocase of inner mitochondrial membrane 10 homolog (yeast)
3.425670081	Ccs	copper chaperone for superoxide dismutase
3.425670081	Mst1r	macrophage stimulating 1 receptor (c-met-related tyrosine kinase)
3.425670081	2610511e22Rik	RIKEN cDNA 2610511E22 gene
3.425670081	Rhot2	ras homolog gene family, member T2
3.425670081	1700013g20Rik	RIKEN cDNA 1700013G20 gene

3.425670081	Tgfa	transforming growth factor alpha
3.425670081	4930455f23Rik	RIKEN cDNA 4930455F23 gene
3.425670081	Bace2	beta-site APP-cleaving enzyme 2
3.425670081	Entpd3	ectonucleoside triphosphate diphosphohydrolase 3
3.425670081	Tcf15	transcription factor 15
3.425670081	Arv1	ARV1 homolog (yeast)
3.425670081	Serhl	serine hydrolase-like
3.425670081	Krt1-13	keratin complex 1, acidic, gene 13
3.425670081	Car11	carbonic anhydrase 11
3.425670081	Dock1	dedicator of cyto-kinesis 1
3.425670081	Ctf1	cardiotrophin 1
3.425670081	Mlh1	mutL homolog 1 (E. coli)
3.425670081	Hsbp1	heat shock factor binding protein 1
3.425670081	3010026o09Rik	RIKEN cDNA 3010026O09 gene
3.425670081	Zfp202	zinc finger protein 202
3.425670081	4930563c04Rik	RIKEN cDNA 4930563C04 gene
3.425670081	1810037k07Rik	RIKEN cDNA 1810037K07 gene
3.425670081	Six5	sine oculis-related homeobox 5 homolog (Drosophila)
3.425670081	Pkp2	plakophilin 2
3.425670081	MGI:2446326	suprabasin
3.425670081	Fbxl4	F-box and leucine-rich repeat protein 4
3.425670081	Gm631	gene model 631, (NCBI)
3.425670081	Olfr181	olfactory receptor 181
3.425670081	Tmlhe	trimethyllysine hydroxylase, epsilon
3.425670081	MGI:1927369	RB-associated KRAB repressor
3.425670081	Chek2	CHK2 checkpoint homolog (S. pombe)
3.425670081	Ralbp1	ralA binding protein 1
3.425670081	Muted	muted
3.425670081	Ccnh	cyclin H
3.425670081	Lefty1	left right determination factor 1
3.425670081	Pxmp2	peroxisomal membrane protein 2
3.425670081	Tloc1	translocation protein 1
3.425670081	Otog	otogelin
3.425670081	3830402i07Rik	RIKEN cDNA 3830402I07 gene
3.425670081	Rdh13	retinol dehydrogenase 13 (all-trans and 9-cis)
3.425670081	Scly	selenocysteine lyase
3.425670081	Leprel2	leprecan-like 2
3.425670081	Bc013529	cDNA sequence BC013529
3.425670081	Evc2	Ellis van Creveld syndrome 2 homolog (human)
3.425670081	Gnai1	guanine nucleotide binding protein, alpha inhibiting 1
3.425670081	Olfr159	olfactory receptor 159
3.425670081	Pms2	postmeiotic segregation increased 2 (S. cerevisiae)
3.425670081	Zfp449	zinc finger protein 449
3.425670081	2310008m10Rik	RIKEN cDNA 2310008M10 gene
3.425670081	Aa407659	expressed sequence AA407659
3.425670081	Spfh2	SPFH domain family, member 2
3.425670081	Rras	Harvey rat sarcoma oncogene, subgroup R
3.425670081	Ssbp1	single-stranded DNA binding protein 1
3.425670081	E130306i01Rik	RIKEN cDNA E130306I01 gene
3.425670081	B430104h02Rik	RIKEN cDNA B430104H02 gene
3.425670081	4632409l22Rik	RIKEN cDNA 4632409L22 gene
3.425670081	Pdrg1	p53 and DNA damage regulated 1
3.425670081	Vps24	vacuolar protein sorting 24 (yeast)
3.425670081	5830454e08Rik	RIKEN cDNA 5830454E08 gene
3.425670081	Fkbp14	FK506 binding protein 14
3.976065312	3110006p09Rik	RIKEN cDNA 3110006P09 gene
3.976065312	MGI:1915703	GPI-anchored HDL-binding protein 1
3.976065312	0710001b24Rik	RIKEN cDNA 0710001B24 gene
3.976065312	Rfng	radical fringe gene homolog (Drosophila)
3.976065312	Col4a2	procollagen, type IV, alpha 2
3.976065312	Cox6a1	cytochrome c oxidase, subunit VI a, polypeptide 1
3.976065312	Pomt2	protein-O-mannosyltransferase 2
3.976065312	Scg3	secretogranin III
3.976065312	Bphl	biphenyl hydrolase-like (serine hydrolase, breast epithelial mucin-associated antigen)
3.976065312	Mtrf1l	mitochondrial translational release factor 1-like
3.976065312	Galnt3	UDP-N-acetyl-alpha-D-galactosamine:polypeptide N-acetylgalactosaminyltransferase 3
3.976065312	Nat2	N-acetyltransferase 2 (arylamine N-acetyltransferase)
3.976065312	2700082d03Rik	RIKEN cDNA 2700082D03 gene
3.976065312	2610040c18Rik	RIKEN cDNA 2610040C18 gene
3.976065312	Snai2	snail homolog 2 (Drosophila)
3.976065312	G630055p03Rik	RIKEN cDNA G630055P03 gene
3.976065312	Crot	carnitine O-octanoyltransferase
3.976065312	Arhgef12	Rho guanine nucleotide exchange factor (GEF) 12
3.976065312	Eva1	epithelial V-like antigen 1
3.976065312	Tsga14	testis specific gene A14
3.976065312	Gpsm2	G-protein signalling modulator 2 (AGS3-like, C. elegans)
3.976065312	Ctbp2	C-terminal binding protein 2
3.976065312	Trpm4	transient receptor potential cation channel, subfamily M, member 4
3.976065312	5730461k03Rik	RIKEN cDNA 5730461K03 gene
3.976065312	Gle1l	GLE1 RNA export mediator-like (yeast
3.976065312	Nsmce1	non-SMC element 1 homolog (S. cerevisiae)
3.976065312	Lyplal1	lysophospholipase-like 1
3.976065312	1190017o12Rik	RIKEN cDNA 1190017O12 gene
3.976065312	Ddit4l	DNA-damage-inducible transcript 4-like
3.976065312	Lpp	LIM domain containing preferred translocation partner in lipoma
3.976065312	Tm7sf2	transmembrane 7 superfamily member 2
3.976065312	Bc011487	cDNA sequence BC011487

3.976065312	1300002c08Rik	RIKEN cDNA 1300002C08 gene
3.976065312	Tbx15	T-box 15
3.976065312	MGI:1914434	genes associated with retinoid-IFN-induced mortality 19
3.976065312	Plscr4	phospholipid scramblase 4
3.976065312	Opn3	opsin (encephalopsin)
3.976065312	C77668	expressed sequence C77668
3.976065312	A830007p12Rik	RIKEN cDNA A830007P12 gene
3.976065312	Cda	cytidine deaminase
3.976065312	Jag2	jagged 2
3.976065312	Ech1	enoyl coenzyme A hydratase 1, peroxisomal
3.976065312	Lgtn	ligatin
3.976065312	Mrpl49	mitochondrial ribosomal protein L49
3.976065312	Nexn	nexilin
3.976065312	Scd3	stearoyl-coenzyme A desaturase 3
3.976065312	Krtap4-7	keratin associated protein 4-7
3.976065312	Kdelr2	KDEL (Lys-Asp-Glu-Leu) endoplasmic reticulum protein retention receptor 2
3.976065312	Bc020002	cDNA sequence BC020002
3.976065312	Cyp20a1	cytochrome P450, family 20, subfamily A, polypeptide 1
3.976065312	Nucb2	nucleobindin 2
3.976065312	1110004e09Rik	RIKEN cDNA 1110004E09 gene
3.976065312	Lig4	ligase IV, DNA, ATP-dependent
3.976065312	2810055f11Rik	RIKEN cDNA 2810055F11 gene
3.976065312	Twist2	twist homolog 2 (Drosophila)
3.976065312	Nudt7	nudix (nucleoside diphosphate linked moiety X)-type motif 7
3.976065312	Rhou	ras homolog gene family, member U
3.976065312	E130303b06Rik	RIKEN cDNA E130303B06 gene
3.976065312	1600002h07Rik	RIKEN cDNA 1600002H07 gene
3.976065312	Bc056485	cDNA sequence BC056485
3.976065312	Phf17	PHD finger protein 17
3.976065312	3110040n11Rik	RIKEN cDNA 3110040N11 gene
3.976065312	Mrp63	mitochondrial ribosomal protein 63
3.976065312	2610002m06Rik	RIKEN cDNA 2610002M06 gene
3.976065312	Mrpl11	mitochondrial ribosomal protein L11
3.976065312	Abhd14a	abhydrolase domain containing 14A
3.976065312	Sptlc1	serine palmitoyltransferase, long chain base subunit 1
3.976065312	Mmp15	matrix metalloproteinase 15
3.976065312	Cap2	CAP, adenylate cyclase-associated protein, 2 (yeast)
3.976065312	Cdkn1c	cyclin-dependent kinase inhibitor 1C (P57)
3.976065312	8430408g22Rik	RIKEN cDNA 8430408G22 gene
3.976065312	Akp2	alkaline phosphatase 2, liver
3.976065312	Elp3	elongation protein 3 homolog (S. cerevisiae)
3.976065312	MGI:1913570	HSPC171 protein
3.976065312	Txnl1	thioredoxin-like 1
3.976065312	Galnt2	UDP-N-acetyl-alpha-D-galactosamine:polypeptide N-acetylgalactosaminyltransferase 2
3.976065312	Bc010787	cDNA sequence BC010787
3.976065312	Tsta3	tissue specific transplantation antigen P35B
3.976065312	Phpt1	phosphohistidine phosphatase 1
3.976065312	Hibch	3-hydroxyisobutyryl-Coenzyme A hydrolase
3.976065312	Ghr	growth hormone receptor
3.976065312	Mtx2	metaxin 2
3.976065312	Cdkl2	cyclin-dependent kinase-like 2 (CDC2-related kinase)
3.976065312	Cfh	complement component factor h
3.976065312	2310046g15Rik	RIKEN cDNA 2310046G15 gene
3.976065312	Znf319	zinc finger protein 319
3.976065312	Bc035537	cDNA sequence BC035537
3.976065312	Actl6a	actin-like 6A
3.976065312	5330431n19Rik	RIKEN cDNA 5330431N19 gene
3.976065312	Adamts18	a disintegrin-like and metalloprotease (reprolysin type) with thrombospondin type 1 motif, 18
3.976065312	M6prbp1	mannose-6-phosphate receptor binding protein 1
3.976065312	Ai427515	expressed sequence AI427515
3.976065312	Mrpl50	mitochondrial ribosomal protein L50
3.976065312	Krtcap2	keratinocyte associated protein 2
3.976065312	Homer2	homer homolog 2 (Drosophila)
3.976065312	Ltbr	lymphotoxin B receptor
3.976065312	Det1	de-etiolated homolog 1 (Arabidopsis)
3.976065312	Slc25a16	solute carrier family 25 (mitochondrial carrier, Graves disease autoantigen), member 16
3.976065312	Dnttip1	deoxynucleotidyltransferase, terminal, interacting protein 1
3.976065312	Fkbp4	FK506 binding protein 4
3.976065312	1810057e01Rik	RIKEN cDNA 1810057E01 gene
3.976065312	A130038l21Rik	RIKEN cDNA A130038L21 gene
3.976065312	Ifitm3	interferon induced transmembrane protein 3
3.976065312	Mrpl10	mitochondrial ribosomal protein L10
3.976065312	Mrpl13	mitochondrial ribosomal protein L13
3.976065312	Hist1h1e	histone 1, H1e
3.976065312	Zbed3	zinc finger, BED domain containing 3

Supplemental Table S2 **Genes with increased expression (FDR < 5%) in control samples in Figure 2A as determined using Significance Analysis of Microarrays (SAM)**

FDR q-value (%)	Gene symbol	Gene name
0	5730557b15Rik	RIKEN cDNA 5730557B15 gene
0	Cxcr4	chemokine (C-X-C motif) receptor 4
0	Clec4e	C-type lectin domain family 4, member e
0	Ptgs2	prostaglandin-endoperoxide synthase 2
0	Arl12	ADP-ribosylation factor-like 12
0	Ccl17	chemokine (C-C motif) ligand 17
0	Nfe2l2	nuclear factor, erythroid derived 2, like 2
0	Gadd45b	growth arrest and DNA-damage-inducible 45 beta
0	Plaur	urokinase plasminogen activator receptor
0	Slc7a11	solute carrier family 7 (cationic amino acid transporter, y+ system), member 11
0	Rel	reticuloendotheliosis oncogene
0	D16ertd472e	DNA segment, Chr 16, ERATO Doi 472, expressed
0	Ltb4r	leukotriene B4 receptor
0	Bc036563	cDNA sequence BC036563
0	Pscdbp	pleckstrin homology, Sec7 and coiled-coil domains, binding protein
0	Itgb7	integrin beta 7
0	Il18rap	interleukin 18 receptor accessory protein
0	Ripk2	receptor (TNFRSF)-interacting serine-threonine kinase 2
0	Csf2	colony stimulating factor 2 (granulocyte-macrophage)
0	Map2k3	mitogen activated protein kinase kinase 3
0	Nfkbie	nuclear factor of kappa light polypeptide gene enhancer in B-cells inhibitor, epsilon
0	Nfkbia	nuclear factor of kappa light chain gene enhancer in B-cells inhibitor, alpha
0	Pmaip1	phorbol-12-myristate-13-acetate-induced protein 1
0	Amn	amnionless
0	Cflar	CASP8 and FADD-like apoptosis regulator
0	Cyld	cylindromatosis (turban tumor syndrome)
0	Prkab2	protein kinase, AMP-activated, beta 2 non-catalytic subunit
0	Apaf1	apoptotic protease activating factor 1
0	Agpat4	1-acylglycerol-3-phosphate O-acyltransferase 1 (lysophosphatidic acid acyltransferase, delta)
0	Bcor	Bcl6 interacting corepressor
0	Olfr90	olfactory receptor 90
0	Tank	TRAF family member-associated Nf-kappa B activator
0	Bc026744	cDNA sequence BC026744
0	Cdkn1a	cyclin-dependent kinase inhibitor 1A (P21)
0	Stk17b	serine/threonine kinase 17b (apoptosis-inducing)
0	5730438n18Rik	RIKEN cDNA 5730438N18 gene
0	Relb	avian reticuloendotheliosis viral (v-rel) oncogene related B
0	D330017j20Rik	RIKEN cDNA D330017J20 gene
0	Hk2	hexokinase 2
0	Il17r	interleukin 17 receptor
0	Krtap16-10	keratin associated protein 16-10
0	Cd274	CD274 antigen
0	1300013d05Rik	RIKEN cDNA 1300013D05 gene
0	2600010e01Rik	RIKEN cDNA 2600010E01 gene
0	Skil	SKI-like
0	Itgal	integrin alpha L
0	Maff	v-maf musculoaponeurotic fibrosarcoma oncogene family, protein F (avian)
0	Fgr	Gardner-Rasheed feline sarcoma viral (Fgr) oncogene homolog
0	Traf1	Tnf receptor-associated factor 1
0	Slc2a3	solute carrier family 2 (facilitated glucose transporter), member 3
0	Slk	STE20-like kinase (yeast)
0	Bc003324	cDNA sequence BC003324
0	Ripk3	receptor-interacting serine-threonine kinase 3
0	Pim1	proviral integration site 1
0	Mefv	Mediterranean fever
0	Serpina3g	serine (or cysteine) proteinase inhibitor, clade A, member 3G
0	Adam23	a disintegrin and metalloprotease domain 23
0.715434784	Aw539457	expressed sequence AW539457
0.715434784	Chi3l3	chitinase 3-like 3
0.715434784	Sfrs15	splicing factor, arginine/serine-rich 15
0.715434784	Bcl10	B-cell leukemia/lymphoma 10
0.715434784	Bcl2a1c	B-cell leukemia/lymphoma 2 related protein A1c
0.715434784	Ptk2b	PTK2 protein tyrosine kinase 2 beta
0.715434784	Sec24a	SEC24 related gene family, member A (S. cerevisiae)
0.715434784	Dnajb6	DnaJ (Hsp40) homolog, subfamily B, member 6
0.715434784	F10	coagulation factor X
0.715434784	Uvrag	UV radiation resistance associated gene
0.715434784	Clcf1	cardiotrophin-like cytokine factor 1
0.715434784	Kif21b	kinesin family member 21B
0.735307972	Slc6a13	solute carrier family 6 (neurotransmitter transporter, GABA), member 13

0.735307972	Ris2	retroviral integration site 2
0.735307972	Glipr1	GLI pathogenesis-related 1 (glioma)
0.735307972	2810451e09Rik	RIKEN cDNA 2810451E09 gene
0.735307972	Il1b	interleukin 1 beta
0.735307972	Gpr35	G protein-coupled receptor 35
0.860848358	C330029b10Rik	RIKEN cDNA C330029B10 gene
0.860848358	Nfkb2	nuclear factor of kappa light polypeptide gene enhancer in B-cells 2, p49/p100
0.860848358	Crem	cAMP responsive element modulator
0.860848358	Cst7	cystatin F (leukocystatin)
0.860848358	Spata2	spermatogenesis associated 2
0.860848358	Arih1	ariadne ubiquitin-conjugating enzyme E2 binding protein homolog 1 (Drosophila)
0.860848358	C630002b14Rik	RIKEN cDNA C630002B14 gene
0.860848358	Snap23	synaptosomal-associated protein 23
0.860848358	F730043m19Rik	RIKEN cDNA F730043M19 gene
0.860848358	Prosc	proline synthetase co-transcribed
0.860848358	Fosl2	fos-like antigen 2
0.860848358	Galnt6	UDP-N-acetyl-alpha-D-galactosamine:polypeptide N-acetylgalactosaminyltransferase 6
0.860848358	Rec8l1	REC8-like 1 (yeast)
1.011633898	Parvg	parvin, gamma
1.011633898	Icam5	intercellular adhesion molecule 5, telencephalin
1.011633898	Zfp364	zinc finger protein 364
1.011633898	A630033h20Rik	RIKEN cDNA A630033H20 gene
1.011633898	1810033b17Rik	RIKEN cDNA 1810033B17 gene
1.011633898	Stk10	serine/threonine kinase 10
1.011633898	Pvr	poliovirus receptor
1.011633898	Tbc1d10c	TBC1 domain family, member 10c
1.011633898	1300013b24Rik	RIKEN cDNA 1300013B24 gene
1.011633898	9430023b20Rik	RIKEN cDNA 9430023B20 gene
1.011633898	Bc034097	cDNA sequence BC034097
1.011633898	Siglec10	sialic acid binding Ig-like lectin 10
1.011633898	Pfkfb3	6-phosphofructo-2-kinase/fructose-2,6-biphosphatase 3
1.011633898	Clec7a	C-type lectin domain family 7, member a
1.011633898	Cias1	cold autoinflammatory syndrome 1 homolog (human)
1.011633898	Card11	caspase recruitment domain family, member 11
1.011633898	Ifrd1	interferon-related developmental regulator 1
1.157205989	Cd52	CD52 antigen
1.157205989	Spn	sialophorin
1.157205989	Pygl	liver glycogen phosphorylase
1.157205989	Edem1	ER degradation enhancer, mannosidase alpha-like 1
1.157205989	Grap2	GRB2-related adaptor protein 2
1.157205989	E2f2	E2F transcription factor 2
1.242774037	Plxnc1	plexin C1
1.242774037	Rapgef6	Rap guanine nucleotide exchange factor (GEF) 6
1.242774037	Cd69	CD69 antigen
1.242774037	Trerf1	transcriptional regulating factor 1
1.242774037	Icoslg	inducible T-cell co-stimulator ligand
1.242774037	Litaf	LPS-induced TN factor
1.242774037	Pdxp	pyridoxal (pyridoxine, vitamin B6) phosphatase
1.242774037	5830405n20Rik	RIKEN cDNA 5830405N20 gene
1.242774037	9130017c17Rik	RIKEN cDNA 9130017C17 gene
1.298581626	Lmnb1	lamin B1
1.298581626	B430306n03Rik	RIKEN cDNA B430306N03 gene
1.298581626	Ptpn22	protein tyrosine phosphatase, non-receptor type 22 (lymphoid)
1.298581626	Mgat4a	mannoside acetylglucosaminyltransferase 4, isoenzyme A
1.298581626	Tob2	transducer of ERBB2, 2
1.298581626	Mtmr4	myotubularin related protein 4
1.298581626	H2-Oa	histocompatibility 2, O region alpha locus
1.298581626	Tlr2	toll-like receptor 2
1.298581626	Ches1	checkpoint supressor 1
1.557122764	Erf	Ets2 repressor factor
1.557122764	Arhgdib	Rho, GDP dissociation inhibitor (GDI) beta
1.557122764	Nfatc2ip	nuclear factor of activated T-cells, cytoplasmic, calcineurin-dependent 2 interacting protein
1.557122764	Fosl1	fos-like antigen 1
1.557122764	Prkx	protein kinase, X-linked
1.557122764	Gch1	GTP cyclohydrolase 1
1.557122764	Adam30	a disintegrin and metalloproteinase domain 30
1.626714843	Tmem39a	transmembrane protein 39a
1.626714843	Lass6	longevity assurance homolog 6 (S. cerevisiae)
1.626714843	Prkcd	protein kinase C, delta
1.626714843	Stk4	serine/threonine kinase 4
1.626714843	Pank2	pantothenate kinase 2 (Hallervorden-Spatz syndrome)
1.626714843	Ccl9	chemokine (C-C motif) ligand 9

1.626714843	Napa	N-ethylmaleimide sensitive fusion protein attachment protein alpha
1.626714843	Serpinc1	serine (or cysteine) proteinase inhibitor, clade C (antithrombin), member 1
1.626714843	Loc223262	similar to small zinc finger-like protein
1.626714843	Cbx4	chromobox homolog 4 (Drosophila Pc class)
1.626714843	2310075c12Rik	RIKEN cDNA 2310075C12 gene
1.626714843	Rcor1	REST corepressor 1
1.895021888	Tnf	tumor necrosis factor
1.895021888	Eif5	eukaryotic translation initiation factor 5
1.895021888	5830446m03Rik	RIKEN cDNA 5830446M03 gene
1.895021888	Zfp263	zinc finger protein 263
2.021069957	Cacnb3	calcium channel, voltage-dependent, beta 3 subunit
2.021069957	Pdcd1	programmed cell death 1
2.021069957	Sesn2	sestrin 2
2.021069957	Slc38a1	solute carrier family 38, member 1
2.021069957	Itpkc	inositol 1,4,5-trisphosphate 3-kinase C
2.188047709	Cry1	cryptochrome 1 (photolyase-like)
2.188047709	Av312086	expressed sequence AV312086
2.188047709	Etf1	eukaryotic translation termination factor 1
2.188047709	5730511k23Rik	RIKEN cDNA 5730511K23 gene
2.188047709	Dna2l	DNA2 DNA replication helicase 2-like (yeast)
2.188047709	2610024e20Rik	RIKEN cDNA 2610024E20 gene
2.188047709	2410025l10Rik	RIKEN cDNA 2410025L10 gene
2.188047709	Bcl2l11	BCL2-like 11 (apoptosis facilitator)
2.188047709	5830408c22Rik	RIKEN cDNA 5830408C22 gene
2.324098412	Lyst	lysosomal trafficking regulator
2.324098412	Pscd1	pleckstrin homology, Sec7 and coiled-coil domains 1
2.324098412	Runx3	runt related transcription factor 3
2.324098412	Csrp3	cysteine and glycine-rich protein 3 (cardiac LIM protein)
2.324098412	Dapp1	dual adaptor for phosphotyrosine and 3-phosphoinositides 1
2.324098412	Il2rb	interleukin 2 receptor, beta chain
2.324098412	Impact	imprinted and ancient
2.324098412	Iqgap1	IQ motif containing GTPase activating protein 1
2.324098412	Gpr162	G protein-coupled receptor 162
2.324098412	Map3k5	mitogen activated protein kinase kinase kinase 5
2.324098412	Bc031781	cDNA sequence BC031781
2.324098412	Gbp5	guanylate nucleotide binding protein 5
2.324098412	Zswim4	zinc finger, SWIM domain containing 4
2.324098412	Sema7a	sema domain, immunoglobulin domain (Ig), and GPI membrane anchor, (semaphorin) 7A
2.324098412	Ikbkb	inhibitor of kappaB kinase beta
2.324098412	4930563d23Rik	RIKEN cDNA 4930563D23 gene
2.324098412	5830461h18Rik	RIKEN cDNA 5830461H18 gene
2.324098412	B4galt5	UDP-Gal:betaGlcNAc beta 1,4-galactosyltransferase, polypeptide 5
2.324098412	Cxcl2	chemokine (C-X-C motif) ligand 2
2.433004319	Trim36	tripartite motif-containing 36
2.433004319	Epsti1	epithelial stromal interaction 1 (breast)
2.433004319	Tnfaip2	tumor necrosis factor, alpha-induced protein 2
2.433004319	Icam1	intercellular adhesion molecule
2.433004319	Cyp4f16	cytochrome P450, family 4, subfamily f, polypeptide 16
2.433004319	Nppb	natriuretic peptide precursor type B
2.433004319	Dyrk1a	dual-specificity tyrosine-(Y)-phosphorylation regulated kinase 1a
2.433004319	Slc15a3	solute carrier family 15, member 3
2.433004319	C530009c10Rik	RIKEN cDNA C530009C10 gene
2.433004319	Marco	macrophage receptor with collagenous structure
2.433004319	Cish	cytokine inducible SH2-containing protein
2.433004319	4930406m16Rik	RIKEN cDNA 4930406M16 gene
2.433004319	Frat2	frequently rearranged in advanced T-cell lymphomas 2
2.433004319	Itgax	integrin alpha X
2.433004319	Dusp2	dual specificity phosphatase 2
2.433004319	Vezf1	vascular endothelial zinc finger 1
2.433004319	Abca7	ATP-binding cassette, sub-family A (ABC1), member 7
2.433004319	Plcg2	phospholipase C, gamma 2
2.433004319	Rfx1	regulatory factor X, 1 (influences HLA class II expression)
2.433004319	Plk3	polo-like kinase 3 (Drosophila)
2.433004319	Emilin2	elastin microfibril interfacer 2
2.433004319	Mef2d	myocyte enhancer factor 2D
2.433004319	9430034d17Rik	RIKEN cDNA 9430034D17 gene
2.433004319	MGI:1298390	SWA-70 protein
2.433004319	Aqp3	aquaporin 3
2.433004319	Bc019206	cDNA sequence BC019206
2.799303861	Bri3bp	Bri3 binding protein
2.799303861	Rnf36	ring finger protein 36
2.799303861	Ifi30	interferon gamma inducible protein 30

2.799303861	F730004d16Rik	RIKEN cDNA F730004D16 gene
2.799303861	2010002n04Rik	RIKEN cDNA 2010002N04 gene
2.799303861	Ear11	eosinophil-associated, ribonuclease A family, member 11
2.799303861	Dpp4	dipeptidylpeptidase 4
2.799303861	Usp38	ubiquitin specific protease 38
2.799303861	Nfe2	nuclear factor, erythroid derived 2
2.799303861	Pole4	polymerase (DNA-directed), epsilon 4 (p12 subunit)
2.799303861	Btbd4	BTB (POZ) domain containing 4
2.799303861	9930117h01Rik	RIKEN cDNA 9930117H01 gene
2.799303861	MGI:2429944	lung-inducible neuralized-related C3HC4 RING domain protein
2.799303861	Chrd	chordin
2.799303861	D730019b10Rik	RIKEN cDNA D730019B10 gene
2.799303861	Kpna4	karyopherin (importin) alpha 4
2.799303861	Slpi	secretory leukocyte protease inhibitor
2.799303861	E130115j16Rik	RIKEN cDNA E130115J16 gene
2.799303861	5830435k17Rik	RIKEN cDNA 5830435K17 gene
2.799303861	Lmna	lamin A
3.425670081	Mcl1	myeloid cell leukemia sequence 1
3.425670081	Pspc1	paraspeckle protein 1
3.425670081	Ddx25	DEAD (Asp-Glu-Ala-Asp) box polypeptide 25
3.425670081	Hbegf	heparin-binding EGF-like growth factor
3.425670081	Prodh2	proline dehydrogenase (oxidase) 2
3.425670081	Fscn1	fascin homolog 1, actin bundling protein (Strongylocentrotus) purpuratus)
3.425670081	4833422f24Rik	RIKEN cDNA 4833422F24 gene
3.425670081	Bmp2k	BMP2 inducible kinase
3.425670081	Cxcl16	chemokine (C-X-C motif) ligand 16
3.425670081	Ifngr1	interferon gamma receptor 1
3.425670081	Map3k14	mitogen-activated protein kinase kinase kinase 14
3.425670081	Tiparp	TCDD-inducible poly(ADP-ribose) polymerase
3.425670081	6530401c20Rik	RIKEN cDNA 6530401C20 gene
3.425670081	Tbx21	T-box 21
3.976065312	Lnpep	leucyl/cystinyl aminopeptidase
3.976065312	5730509c05Rik	RIKEN cDNA 5730509C05 gene
3.976065312	Olr1	oxidized low density lipoprotein (lectin-like) receptor 1
3.976065312	Atp4a	ATPase, H+/K+ transporting, alpha polypeptide
3.976065312	6330407g11Rik	RIKEN cDNA 6330407G11 gene
3.976065312	Ms4a6c	membrane-spanning 4-domains, subfamily A, member 6C
3.976065312	Gvin1	GTPase, very large interferon inducible 1
3.976065312	Lrrk1	leucine-rich repeat kinase 1
3.976065312	MGI:1351468	cytokine-dependent hematopoietic cell linker
3.976065312	Amica1	adhesion molecule that interacts with CXADR antigen 1
